# A Review of In Vivo and Clinical Studies Applying Scaffolds and Cell Sheet Technology for Periodontal Ligament Regeneration

**DOI:** 10.3390/biom12030435

**Published:** 2022-03-11

**Authors:** Maria Bousnaki, Anastasia Beketova, Eleana Kontonasaki

**Affiliations:** Department of Prosthodontics, School of Dentistry, Faculty of Health Sciences, Aristotle University of Thessaloniki, GR-54124 Thessaloniki, Greece; mbousnaki2689@hotmail.gr (M.B.); anastasiabeketova@yahoo.com (A.B.)

**Keywords:** periodontal ligament, periodontal regeneration, cell-sheet technology, in vivo animal models, clinical trials, scaffolds, growth factors, stem cells, patterning, implants

## Abstract

Different approaches to develop engineered scaffolds for periodontal tissues regeneration have been proposed. In this review, innovations in stem cell technology and scaffolds engineering focused primarily on Periodontal Ligament (PDL) regeneration are discussed and analyzed based on results from pre-clinical in vivo studies and clinical trials. Most of those developments include the use of polymeric materials with different patterning and surface nanotopography and printing of complex and sophisticated multiphasic composite scaffolds with different compartments to accomodate for the different periodontal tissues’ architecture. Despite the increased effort in producing these scaffolds and their undoubtable efficiency to guide and support tissue regeneration, appropriate source of cells is also needed to provide new tissue formation and various biological and mechanochemical cues from the Extraccellular Matrix (ECM) to provide biophysical stimuli for cell growth and differentiation. Cell sheet engineering is a novel promising technique that allows obtaining cells in a sheet format while preserving ECM components. The right combination of those factors has not been discovered yet and efforts are still needed to ameliorate regenerative outcomes towards the functional organisation of the developed tissues.

## 1. Introduction

Periodontitis is a bacteria-driven infectious oral condition that can lead to severe degeneration of periodontal tissues with high prevalence (42.2%) in adults aged 30 years and older [1]. In severe conditions, bone tissue destruction occurs in forms of crater-like defects around teeth roots. These intrabony defects constitute a major challenge to periodontal disease treatment; they can be treated through conventional root scaling and planning healing but without the formation of new supporting tissue [2], while remaining periodontal pockets can cause aesthetic problems and act as triggers to further destruction. Currently, the outermost goal of periodontal therapy is the simultaneous regeneration of all periodontal tissues, i.e., new alveolar bone, cementum, and periodontal ligament (PDL). Towards this direction, surgical treatment is based on the Guided Tissue Regeneration (GTR) approach, during which appropriate membranes are utilized to protect bone defect from epithelial tissue downgrowth, allowing the healing and regeneration of the underlying tissues [3]. Although GTR provides improvement over conventional open flap surgery, there are many factors that can considerably affect the clinical outcomes and as shown in a recent systematic review and meta-analysis, the improvement may not be statistically significant [4], and large observational studies are still needed to clarify the exact role of these factors to facilitate dental professionals to safely apply this technique according to patient specific conditions and demands.

Many studies have attempted to engineer a suitable environment for periodontal tissue regeneration, by applying the appropriate regulatory signals, progenitor cells, extracellular matrix (ECM) or carrier constructs and adequate blood supply, needed to regenerate all periodontal tissues, bone, cementum, and PDL [5,6,7,8,9]. Although the majority of studies have dealt predominantly with bone regeneration and in clinical practice most surgical approaches are based on guided bone regeneration, recent efforts have focused on the regeneration of PDL, along with bone and cementum [10,11,12,13]. The perpendicular alignment of new highly organized collagen fibers, inserted into the regenerated cementum and bone, is the most fundamental aspect of the whole periodontal tissue complex regeneration and emerging efforts are dedicated to this ultimate goal.

### 1.1. Periodontal Tissues

#### 1.1.1. Periodontal Ligament 

Periodontal ligament is a fibrous connective tissue lying between alveolar bone and root cementum, occupying a space of 100 to 400 μm. It originates from neural crest-derived ectomesenchyme and is characterized by large heterogeneity in cell populations [14], extensive blood supply [15], and neural network [16]. Development of PDL starts with root formation, before tooth eruption [17,18]. Root formation starts after the formation of enamel and dentin in the area of the future cementoenamel junction, with the Hertwig’s epithelial root sheath (HERS) that is formed by the inner and outer enamel epithelium of the enamel organ. HERS is responsible for the shape and number of tooth roots and induces dentin formation from odontoblasts [18]. After root dentin starts to form, HERS is disintegrated and loses attachment with tooth root. However, its remnants are still present in the form of epithelial cell of Mallasez. By HERS disintegration, dental follicle cells (DFCs) come into contact with newly formed dentin, inducing the formation of cementoblasts that start to secrete cementoid tissue, which is then mineralized to cementum. The initiating factor for cementogenesis is the deposition from the HERS cells of enamel matrix proteins on the root surface [19,20]. Cells of the dental follicle differentiate into fibroblasts, which are responsible for the production of PDL fibers. PDL fibers start to grow from both cementum and alveolar bone, and they gradually elongate during tooth development. In the first steps of PDL formation, collagen fibers are loosely configured near cementum and run in parallel with the root of the teeth [21]. The direction of the fibers changes during toot eruption and appears to be affected by the position of the adjacent teeth. When teeth show up in the oral cavity, dento-gingival, transseptal, and alveolar crest fiber groups appear, while after occlusal contact fibers become apparent and in the apical third of the root [22]. Dense Sharpey’s fibers appear to emerge from the alveolar bone in the cervical part of the root and extend towards thin cementum anchored fibers occupying the PDL space. They get thicker, are organized in distinct bundles, and gain their final dimensions and orientation after full occlusal function of teeth [23]. The basic characteristic of these fibers is that they are enclosed within cementum and bone, and this is particularly important for the regeneration of PDL. 

The PDL fibrous matrix consists of collagen, reticulin, and oxytalan fibers. The 90% of PDL fibers are collagenous, primarily of type I collagen. They provide the structural strength of PDL, while oxytalan fibers that grow during the development of root and the vascularization network in the PDL, seem to play a role in vascular support [24]. It has been reported that HERS, and cementoblasts have a role in oxytalan fibers development during root development, explaining their closer proximity with cementum [25]. Based on their position and orientation PDL fibers are categorized as alveolar crest, apical, horizontal, oblique, and interradicular fibers which lie between the roots of multirooted teeth. 

Different cell populations co-exist in PDL [26]. They are mainly divided in two different lineages, one including basically fibroblastic type cells and another including cells being responsible for mineralized tissue production such as osteoblasts. Although, the predominant cell type is the PDL fibroblast [27], other cell types such as osteoblasts, osteoclasts, fibroblasts, epithelial rests of Malassez, cementoblasts, macrophages, endothelial cells, neural cells and undifferentiated mesenchymal cells (MSCs) being the precursor cells for bone, PDL proper, and cementum have been identified in PDL [18,28,29,30,31]. Numerous studies have shown the potential of PDL-derived cells to differentiate toward a chondrogenic, osteoblastic, angiogenic, adipogenic, and neurogenic phenotype upon appropriate culture conditions and inductive media [32,33,34,35,36,37,38,39,40]. Due to the large heterogeneity of PDL-derived cells, and the need for isolation of multipotent PDL cells that could be effectively applied in PDL regeneration, primary cells are characterized in terms of expressing certain PDL marker genes such as periostin, S100A4 and periodontal ligament-associated protein-1 (PLAP-1), scleraxis, and tendon [30,41,42], and markers similar to bone mesenchymal stem cells. Seo et al [43,44] have shown that an early well-known stem cell marker, STRO-1, is also an early progenitor marker for periodontal ligament stem cells (PDLSCs). They also found that PDLSCs express STRO-1/CD146 markers similar to mesenchymal stem cells. Numerous studies have also shown the capability of PDLSCs to express other MSCs markers such as CD10, CD13, CD29, CD44, CD59, CD73, CD90, CD166, and CD105 [44,45,46,47,48]. At the same time, PDLCSs do not express the hematopoietic progenitors cell markers CD14, CD34, CD45, and HLA- DR, verifying their somatic nature [47]. 

PDL fibroblasts are responsible for the production and maintenance of the extracellular matrix, which is mainly composed of fibers [49]. These cells synthesize and digest fibrillar collagen and produce various bioactive compounds related to wound healing and remodeling [50]. PDL fibroblasts have the capacity to withstand and dissipate the high occlusal loads exerted upon mastication and thus act as a mechanical load absorbing device [51]. Apart from protection against high mastication forces, together with the gingival tissues, PDL forms an effective shield against oral bacteria [52].

#### 1.1.2. Cementum

PDL regeneration requires deep understanding of the hierarchical complexity of dental cementum, as cementum is a basic component of periodontal attachment apparatus that provides anchoring of the principal collagen fibers of the PDL to the root surface. In addition, cementum has an important role in PDL regeneration, as its components and specific microtopography tailor the responses of PDL cells [53]. Cementum is a natural composite containing inorganic hydroxyapatite (HA) nanocrystals and organic matrix rich in collagen fibers (predominantly type I collagen), while non-collagenous matrix proteins like proteoglycans, acidic glycoproteins, growth factors, and attachment proteins occupy the interfibrillar spaces. There are three types of cementum [54]. Acellular cementum covers the cervical two-thirds of the root surface and has a thickness ranging from 50 to 200 μm [55,56]. Its main function is to anchor tooth through periodontal ligament fibers (Sharpey’s). It contains cell-free mineralized matrix, densely packed and radially oriented collagen fibers. The apical portion of the root and the furcation areas are generally layered by cellular mixed stratified cementum. Cellular cementum is characterized by a stratified structure with intrinsic and extrinsic collagen fibers. Extrinsic collagen fibers are derived from PDL, while intrinsic contain entrapped cementocytes. Acellular afibrillar cementum is a type of acellular cementum usually found along the cementoenamel junction [57]. It has a thickness of ~15 μm and is composed of a matrix with mineralized glycosaminoglycans, but without either cementoblasts or collagenous fibers. Ideally, in periodontal engineering approaches, efforts should be made to regenerate cement-like tissue as close as possible to acellular extrinsic fiber cementum, because that type of cementum is the most appropriate to ensure attachment [55,57]. 

### 1.2. Cell-Guided PDL Regeneration

Periodontal ligament (PDL) regeneration is a challenging and ambitious task, since it demands a highly coordinated spatiotemporal healing procedure, which includes bone formation within the periodontal defect, along with cementogenesis and PDL fiber formation and attachment on to the root surface [58]. Additionally, challenges arise from the avascular nature of the tooth surface, by the bacterial accumulation along with the technically challenging operating environment due to limited access [58]. Tissue engineering has emerged recently, targeting to potentially regenerate various tissues and organs, including the periodontium [59]. A tissue engineering approach encompasses the use of 3D scaffold, combined with bioactive molecules and cells, and has the potential to regulate the healing process and bypass the abovementioned challenges [59]. 

As cell-based PDL regeneration attempts have increased over the last years, the application of post-natal progenitor cells has risen, making them an attractive choice for tissue engineering applications. MSCs present the most extensively applied cell type for cell-based regeneration, due to their multi-differentiation capacity, immunomodulation, anti-apoptosis, angiogenesis, and cell recruitment [60]. Different cell types have been applied in previous attempts of cell-based periodontal regeneration, such as bone marrow MSCs (BMMSCs), PDLSCs, gingival fiborblasts (GFs), and dental pulp stem cells (DPSCs).

PDL stem cells (PDLSCs) are the cells that have been isolated from the PDL, and possess characteristics similar to those of MSCs, and a unique potential to regenerate complex PDL tissues [61]. Gingival fibroblasts (GF), which are the most common cell type present in the gingival tissue, are known to modify their behavior and translocate into periodontal defects [62]. It has been found that GF have the ability to form mineralized tissue and express bone-related proteins, while they have been used in regenerative applications, reinforcing the hypothesis that GF possess stem cell characteristics [62]. BMMSCs have demonstrated the ability to proliferate extensively and to differentiate into multiple cell lines; however, their application in periodontal defects has provided some contradictory results [63]. Their effectiveness has been directly related to the morphology of the defect, where increased bone formation has been documented in fenestration and grade III furcation defects, whereas BMSCs application in three-wall intrabony defects had limited effects on new bone formation [63]. Dental pulp stem cells (DPSCs) have also emerged as a potential cell source for tissue engineering applications, as they are easily accessible, can be obtained in large numbers in a non-invasive way, and have multilineage differentiation potential [64]. DPSCs administration has been considered as a possible treatment strategy and DPSCs have been applied in vivo targeting periodontal regeneration. Although their application might be beneficial in terms of bone regeneration, their effectiveness regarding cementum or PDL regeneration is still questionable [65].

Nonetheless, tissue engineering applications do not always render the desired results, due to the immune response triggered by degradation of the scaffolds [58]. Additional problems that arise are low survival of expanded and grown cells in vitro before implantation into the living body, inability of injected cells to attach to the site of implantation, and lack of vascularization or difficulty in revascularization in the site of interest [66,67]. Techniques that utilize extracellular matrix (ECM) production in vitro prior to cell transplantation, such as cell sheets or cell pellets, have gained attention in attempts to overcome the problems encountered by the tissue engineering strategies.

**Figure 1:**
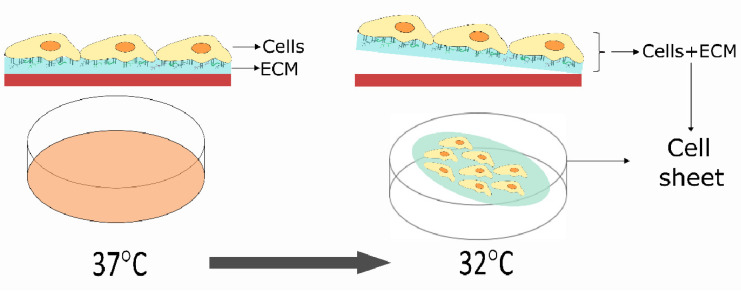
Schematic drawing of cell sheet harvesting.

Cell sheet engineering is a novel technique that allows the acquirement of cells in a sheet format, without the application of proteolytic enzymes or other disruptive method, thus enabling the preservation of ECM components [68] (Figure 1).

Different methods have been employed to harvest cell sheets, such as the use the temperature responsive culture dishes, the use of polymerized human fibrin-coated dishes, and the use of Vitamin C (Vc) treatment [69]. The use of temperature-responsive culture dishes was the first method applied to obtain cell sheets, and has been the most extensively implemented, with the utilization of Poly(N-isopropylacrylamide) (PIPAAm), which is a temperature responsive polymer [70]. A smart biointerface from PIPAAm was developed, which allowed the control of cells attachment through the manipulation of temperature [71]. In normal cell culture conditions of 37 °C, the surface remains hydrophobic, allowing cells to attach and proliferate, and changes into hydrophilicity below the critical tempeature of 32 °C, leading to cells detachment from culture surface without the use of proteolytic enzymes [70]. This technique exhibits numerous advantages over conventional methods, as the cells preserve the integrity of adhesion proteins, such as E-cadherin and laminin 5, retain its ECM components secreted by the cells, and have minimal cell loss [70]. Cell sheets can be directly applied into a defect area either as a coating, as numerous cell sheets can overlap each other, creating a three-dimensional structure, or even shrink and create a cell pellet, that can be applied as a graft into the area of interest.

The aim of this review was to discuss recent advancements and strategies for PDL regeneration in terms of clinical outcomes derived from in vivo models and clinical studies, by applying cell sheet technology and scaffold constructs. A search strategy was applied to include most of the available literature in Web of Science, Pubmed and Scopus databases. Search terms included “periodontal”, “periodontal ligament”, “regeneration”, “in vivo”, “scaffolds”, “clinical trial”, “clinical study”, “clinical”, “stem cells”, “progenitor cells”, “precursor cells”, “pluripotent stem cells”, “multipotent stem cells”, “embryonic stem cells”, “ips cells”, “somatic cells”, “mesenchymal stem cells”. Hand searching from selected review articles and other included articles was also performed. 

## 2. Included Studies

### 2.1. In Vivo Studies

**Figure 2:**
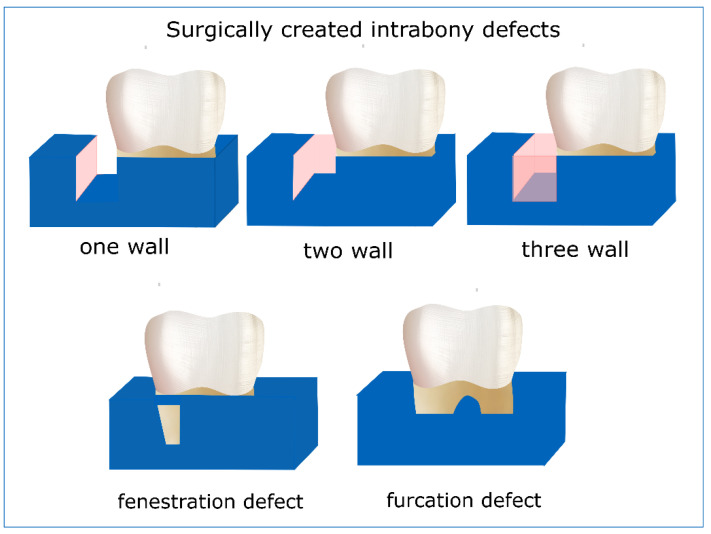
Most common employed periodontal defect models for periodontal tissue regeneration.

The included in vivo studies employed the intrabony/furcation periodontal defect model or the fenestration periodontal defect mode (Figure 2) as an orthotopic model and different ectopic models to test the regenerative capacity of tissue engineering constructs consisting of scaffolds, matrices, membranes, hydrogels etc., either loaded with growth factors or seeded with different cells/cell sheets.

#### 2.1.1. Cell Sheet Engineering

##### Ectopic Models

Twenty-six studies used ectopic models to assess the periodontal regenerative capacity of cell sheet transplantation (Supplementary Table S1). Twenty-five of these studies used nude mice, while one study used rats. The included studies used a variety of biomaterials in an attempt to simulate the orthotopic conditions and ectopically assess the potential for periodontal regeneration of each cell sheet, such as ceramic bovine bone (CBB), chemical conditioned root dentin (CCRD), dentin block, polyglycolic acid (PGA) film, gelfoam scaffold, treated dentin matrix (TDM), hydroxyapatite/tricalcium phosphate (HA/TCP), titanium (Ti), teeth roots, platelet-rich fibrin (PRF) fabricated into bioabsorbable fibrin scaffolds, decalcified dentin matrix (DDM), polycaprolactone (PCL) scaffold, Matrigel, and micro/macro-porous biphasic calcium phosphate (MBCP) blocks. Two studies implanted the cell sheet/material complex into jawbone implant sockets, using bioengineered tooth root (bio-root) structure from HA/TCP, wrapped with the cell sheet [72,73]. Furthermore, two studies assessed the regenerative potential of cell sheets combined with titanium samples [66,74]. In the study by Washio et al. [66], hPDLCs sheets/titanium implant complexes were transplanted into mandibular bone defects, were histological observation demonstrated the formation of cementum and PDL-like tissue on titanium surface. Those findings support the prospect of future efforts towards the formation of a stable periodontal complex around dental implants.

To produce the cell sheets, a variety of cells were applied in the different studies using ectopic models. Most of the included studies (20 studies) formed cell sheets from PDLSCs [72,73,74,75,76,77,78,79,80,81,82,83,84,85,86,87,88,89,90,91], followed by dental follicle stem cells (DFCs) (five studies) [83,84,92,93,94], PDLCs (four studies) [66,95,96,97], BMMSCs (two studies) [74,77], jaw BMMSCs (JBMMSCs) (two studies) [78,87], and (DPSCs (two studies) [82,98], while the following cells, osteoblastic cells [97], apical tooth germ cells (APTGs) [76], human umbilical vein endothelial cells (HUVEC) [89], stem cells from the apical papilla (SCAP) [82], urine-derived stem cells (USCs) [88], stem cells from human exfoliated deciduous teeth (SHEDs) [94] were used in one study each. 

Several different pretreatments were used in the included studies with intendence to enhance the regenerative potential of cell sheets. Five studies used the Vc pretreatment as the method of choice for the cell sheet fabrication [72,73,75,82,98]. Li et al. [91] assessed the effect of low-intensity pulsed ultrasound (LIPUS) stimulus on PDLSC sheet formation and periodontal tissue regeneration in vivo. Their results highlighted the positive effect of LIPUS-treated PDLSC sheets on ECM synthesis and PDL-like tissue regeneration compared with the untreated PDLSC sheet group. The effect of pretreatment of human PDLSC (hPDLSC) sheets with recombinant human bone morphogenetic protein-2 (rhBMP-2) targeting the regeneration of dental cementum and the periodontal complex was evaluated in an ectopic model of nude mice [81]. Pretreated hPDLSC sheets exhibited significantly more mineralization and collagen ligament accumulation as compared with the control group, thus enabling the formation of PDL cementum-like complex [81]. Yang et al. [76] assessed the effect of conditioned medium (CM) from developing apical tooth germs on hPDLSC sheets, that were then transformed into cell pellets to be used for periodontal tissue engineering. Cementum-like mineralized tissues and PDL-like fibrous tissues were identified in the CM treated group, whereas control group cultured without CM rarely formed cementum/PDL-like tissue [76]. Platelet-rich derivatives were used in two studies, one used platelet-rich plasma (PRP) as pretreatment, while the study by Wang et al. used platelet-rich fibrin (PRF) as a bioabsorbable scaffold [78,85]. PRP pretreatment resulted in significantly enhanced osteogenic differentiation of PDLSCs and increased bone and collagen formation in vivo compared with untreated control [85]. Moreover, the use of PRF as a bioabsorbable scaffold was more beneficial in terms of PDL and bone tissue formation when combined with jaw BMMSC instead of PDLSC sheets [78].

Five studies assessed the effect of coculture of different cells on the properties and effectiveness of cell sheets, as well as their regenerative abilities [77,84,87,88,89]. Coculture of PDLSCs with a different cell line seems to be beneficial on the properties of the cell sheet that results from the coculture system. More specifically, hPDLSCs were cocultured with hBMMSCs, and the mixed cell sheet was used to create a cell pellet which was applied in vivo for ectopic transplantation, showing enhanced cementum/PDL-like tissue regeneration with neovascularization when compared to the non-mixed cell pellet [77]. Furthermore, the in vivo application of cell sheets from the coculture of PDLSCs with either urine-derived stem cells (USCs) or jaw BMMSCs resulted in increased expression levels of bone- and ECM-related genes and proteins and led to the formation of a complex tissue like the native periodontal tissue [87,88,89]. Panduwawala et al. fabricated triple-cell sheets from PDLSCs and human umbilical vein endothelial cells (HUVECs), either from combination of the different cell sheets (PDLSCs-HUVECs-PDLSCs) or cell sheets from the coculture of these cells and found that both conditions resulted in periodontal fiber formation similar to PDL, as well as vascular lumen formation [89]. Liu et al. assessed the regenerative capacity of PDLSCs from healthy subjects (HPDLSCs) and patients diagnosed with periodontal disease (PPDLSCs) when cocultured with DFCs [84]. DFCs seem to enhance the stemness of both HPDLSCs and PPDLSCs, and the cocultured HPDLSC sheet managed to regenerate the PDL complex, whereas in the case of PPDLSC sheet, fibers did not adhere well while inflammatory cells were also present in the regenerated tissue [84].

##### Orthotopic Models

Twenty of the studies used orthotopic models to assess the regenerative capacity of cell sheet transplantation in periodontal defect models, with or without biomaterials (Table 1). 

The studies investigated the potential of cell sheets towards periodontal tissue regeneration in vivo through various animal models and experimental strategies. Different periodontal defect models were used, where two studies used one-wall bone defect model [94,101], two studies used three-wall intrabony defects [104,105], and each of the following defect models were used in one study, a dehiscence defect model [110], a class III furcation defect model [92], a two-wall intrabony defect model [107], a horizontal defect model [106], and a fenestration defect model [99], while the rest of the studies did not specify the morphology of the defect. Six studies assessed cell sheet application in a rat model [86,94,99,103,109,112], six studies used dogs as the animal model of choice [101,102,106,107,108,111], four studies used miniature pigs [75,100,104,105], two studies used rats [90,97], one study used sheep [110], while there was also 1 clinical study [8].

To produce the cell sheets, the different orthotopic studies used a variety of cells. Most of the included studies (eight studies) formed cell sheets from PDLSCs [75,86,90,100,101,102,103,106], followed by PDLCs (six studies) [8,97,107,109,110,112], BMMSCs (three studies) [101,108,110], and dental follicle stem cells (DFCs) (three studies) [94,107,111], gingival fibroblasts (two studies) [99,110], DPSCs (two studies) [104,105], while the following cells, osteoblastic cells [97], alveolar periosteal cells (APCs) [101], and stem cells from human exfoliated deciduous teeth (SHEDs) [95], were used in one study, each. Tsumanuma et al. [101] assessed the effect of three-layered cell sheets from different cell lines in one-wall surgically created defects in dogs and found that the application of PDLC sheets resulted in more newly formed thick acellular/cellular cementum, denser collagen fibers and enhanced PDL formation compared to the BMMSC and APC sheets groups. In the study by Guo et al. [107], the effectiveness of DFC sheets and PDLC sheets towards periodontal regeneration was assessed in a two-wall intrabony defect in dogs. While new periodontal attachment was observed in both groups, complete periodontal regeneration involving PDL and cementum was detected only in the DFC sheet group, which also exhibited enhanced bone formation when compared to the PDLC sheets. Whereas the study by Yang et al. showed similar periodontal regeneration potential between DFC sheets and SHED sheets [94]. More specifically, the regenerated tissues observed in both experimental groups were all consisting of fibroblasts and collagen fibers, which were perpendicularly arranged and well organized, similar to that of native PDL [94]. Another study showed the superiority of the application of a complex cell sheet containing two cell lines, PDLCs and osteoblastic cells, against the application of each single cell sheet containing either cell line [97]. In detail, complex cell sheet application resulted in new bone formation and complete PDL regeneration, restoring the functional connection between the alveolar bone and tooth root, whereas control groups exhibited incomplete recovery in both mineralized tissue and soft tissue formation [97]. When comparing three different cell sheets in a surgically created dehiscence periodontal defects in sheep, Vaquette et al. found that BMMSC and PDLC sheets demonstrated similar results in terms of new bone formation, PDL and cementum regeneration after 10 weeks, whereas both groups exhibited superior regenerative potential when compared to GF sheets [110].

Wei et al. assessed different methods for the obtainment of cell sheets, the use of temperature responsive culture dishes and the application of Vc, and its effect on periodontal regeneration potential of PDLSC sheet [75]. Vc-induced PDLSC sheets application into the defect area resulted in increased bone/cementum-like matrix formation, which was significantly higher compared to the PDLSC sheets from the temperature responsive culture dishes [75]. Two studies assessed the effect of different pretreatments, such as inflammatory stimulation or hypoxia, on the regenerative potential of cell sheets [90,107]. The study by Yu et al. showed that 24-hour hypoxic pretreatment of PDLSCs enhanced their regenerative potential in vivo in terms mineralized tissue and cementum formation, and PDL regeneration [90].

The cell sheets in the different studies were combined with a variety of biomaterials, such as HA/TCP, CBB, TDM, Matrigel, gel foam scaffold, platelet-rich fibrin granules, polycaprolactone scaffold, polyglycolic acid (PGA), and porous β-TCP. 

The effect of platelet-rich fibrin granules on PDLSCs sheet fragments targeting periodontal regeneration was assessed in the study by Zhao et al. [102]. In the tooth reimplantation model used in this study, the combined application of PDLSC/PRF was more effective in regenerating PDL-like tissues and avoiding ankylosis and inflammation, compared to the other groups [102].

Iwasaki et al. used a decellularized amniotic membrane (amnion), instead of an engineered cell sheet, with or without PDLSCs in a surgically created periodontal defect in rat maxillary molars; and found that the presence of PDLSCs enhanced periodontal tissue regeneration four-weeks post-transplantation, as indicated by the radiological and histological analysis [103]. In the study by Jiang et al., decellularized sheets from human PDL cells were combined with 15-deoxy-Δ12,14-prostaglandin J2 (15d-PGJ2) nanoparticles along with or without polycaprolactone/gelatin (PCL/GE) nanofibers as potential candidates for periodontal regeneration in rat periodontal defect model [112]. The application of decellularized hPDLCs sheets resulted in successful bone tissue ingrowth, as well as cementum-like and PDL-like tissue formation on the root the mandibular first molar, despite the presence or absence of PCL/GE nanofibers [112]. Farag et al. [109] assessed the effect of decellularized PDLSCs sheet combined with PCL scaffold on periodontal regeneration in a rat periodontal defect model, where the beneficial role of the decellularized matrix was demonstrated. More specifically, the decellularized sheets were infiltrated with cells, and exhibited significantly higher new attachment of periodontal fibers when compared with the PCL scaffolds alone, while the regenerated PDL fibers were more organized and inserted with a perpendicular allignemnt into the root surface [109]. In the study by Yang et al. [111], the use of TDM particles or HA/TCP combined with DFCs resulted in increased bone formation when compared to the groups without materials. Furthermore, the presence of DFCs had a positive effect on the density of bone formation and the extent of PDL-like tissue formation compared to the control group without cells [111].

### 2.2. In Vivo Studies with Scaffolds for PDL Regeneration

A large variety of biomaterials in the form of simple, biphasic, or multiphasic scaffolds have been proposed for the regeneration of damaged periodontal tisses. These 2D and 3D constructs have been developed based on concepts of complete regeneration of the periodontal apparatus (bone, PDL, and cementum) or partial regeneration of specific compartments, such as PDL or bone/cementum tissues, with most of them investigating the osteogenic capacity of their materials focusing on bone regeneration. As GTR still constitutes the “gold standard” in periodontal surgical interventions, various degradable or non-degradable membranes (2D structures) have been utilized to prevent epithelium downgrowth to allow a smooth healing of damaged periodontal connective tissue and ligament. The use of scaffolds aims to develop biocompatible and bioactive platforms that, with the help of other attached or loaded molecules and growth factors, can lead to timely and guided cell migration, proliferation, and differentiation to promote tissue regeneration. Towards this direction, different animal species (dogs, miniature pigs, rats, etc.) and in vivo models have been evaluated, including scaffolds placement in surgically created periodontal defects or ectopic tissue formation by subcutaneous implantation in animals. 

#### 2.2.1. Periodontal Defect Model

Most in vivo studies evaluating scaffolds employed the intrabony/furcation periodontal defect model or the fenestration periodontal defect model (Table 2). Surgical creation of one- or two-wall bone defects near the roots of molars and premolars, PDL and cementum removal and scaffold placement in close proximity to root dentin, are the major steps for the furcation model, while defects with standardized height (usually 5 mm in apico-coronal direction at the furcation region) are created around premolars or molars in the furcation model. 

Beagle dogs have been widely used in studies evaluating various therapeutic strategies for periodontitis and the regeneration of periodontal tissues with scaffolds [137]. The rational for their use lies in the similarities of their periodontal tissues’ architecture and oral microflora with humans [138]. In addition, proper hygiene can be achieved without sedating the animals, which ensures animals convenience and low risk of complications to proper healing and regeneration [139,140]. Significant limitations of dog models are that dogs do not exert lateral movements during mastication and that they present greater bone remodeling rate that could yield high regenerative potential and subsequently to optimized results of in vivo studies [141,142]. Two different surgical protocols are used; one comprising the creation of supraalveolar critical-size furcation defects and the other creating intrabony defects [143]. Hydrogels [13,125] composite collagen-hydroxyapatite scaffolds [123], bioceramic [116,144] and bioactive glass scaffolds [122], nanoparticles-loaded polymeric or collagen scaffolds [124], and combined micropatterned polymeric scaffolds [130], either cell loaded or not, have been tested in the beagle or other dog models. The whole periodontal complex was regenerated, with obliquely inserted ligament-like fibers when a biphasic scaffold consisting of gelatin and β-TCP/HA particles (BH) and biphasic cryogel scaffold (BCS) was implanted in beagle dogs, loaded with BMP-2 and protected by a functionally graded membrane [13]. The use of FGF-2 was advantageous in combination with a nano-β-TCP collagen scaffold for the development of accelular cementum on the surface of exposed roots and the formation of PDL-like tissue [124]. Similar were the findings of Momose et al [125], with the use of a collagen hydrogel scaffold loaded with FGF-2; however, both studies although verified the presence of PDL-like fibrous tissue, they did not observe tissue attachment and functional Sharpey’s fibers formation. Bioceramic diopside ceramics proved more efficient in producing large quantities of bone, cementum and well-oriented collagen fibers compared to β-TCP, that presented only limited new bone or osteoid deposition [144]. Cell-loaded chitosan/anorganic bovine bone composite scaffolds and collagen sponges presented greater volumes of new bone and cementum, with dense PDL fibers [115], in an oblique or perpendicular orientation [127]. On the other hand, limited positive effect was reported from Liu et al, that used collagen-hydroxyapatite scaffolds loaded with BMSCs, possibly explained from the limited survival of cells within the scaffold due to the poor blood supply of labial alveolar bone [123]. 

Miniature pigs have been used as a more convenient and reliable animal model in many studies in dentistry, but surprisingly very few studies exist on testing scaffolds for PDL regeneration in miniature pigs [113,118]. Pigs’ bone anatomy and morphology, healing, and rate of remodeling are considered to be close to those of humans, and therefore is a suitable animal species, as evidenced from a lot of studies in recent years [137]. In addition, pigs have anatomically and functionally temporomandibular articulation close to that in humans and as omnivores, they masticate with lateral jaw movements, representing a more suitable model for mimicking the mastication cycle [145]. However, they have inherent limitations, such as larger teeth surrounded of large bone volume, long junctional epithelium, and different oral microflora. Only a few studies have used the periodontal defect model in the miniature pig in combination with scaffolds for PDL regeneration [113,118]. Hybrid tooth constructs from PGA/PLLA and PLGA scaffolds for tooth and bone parts respectively were seeded with DSCs and remained for 12 and 20 weeks [113]. Despite the new cementum formation, periodontal ligament fibrous tissue resembling natural Sharpley’s fibres was found scarcely. A hyaluronic hydrogel scaffold releasing IL-1-resceptor antagonist was used in an effort to optimize regeneration by restricting the inflammatory stage of periodontal wound healing [118]. Although bone and cementum-like tissue were formed and PDL-like fibers were anchored to cementum, no distinct effect was observed in the group with the IL-1-resceptor antagonist.

Rat species is the most used animal model implementing the periodontal defect model in recent articles. Despite rats presenting continuous teeth eruption and periodontal remodeling with cementum and bone apposition, that can yield optimized results in respect to potential regenerative materials and approaches, their ease of handling and low maintenance cost, along with low ethical or social concern, have made them prevail in periodontal tissue regeneration studies (Table 2). In general, the rat periodontal fenestration defect model with an extraoral (buccal) surgical approach is used and is widely adapted as a valid model before proceeding to larger animal testing. The advantage of this approach is that the chance of gingival tissue ingrowth is eliminated, however it is technically more demanding [143]. Athymic nude, Sprague-Dawley, Fischer 344, and Wistar rats have been used in different studies in combination with varying scaffolding materials for PDL regeneration. Hydrogel scaffolds [128,131,133] and various fibrous polymeric constructs like PCL [12,109,114,132], PLGA/PCL [122,130], PCL/PEG [120], and PLGA [136] scaffolds or membranes have been implanted in periodontal defects in rats, fabricated mostly by electrospinning. A biomimetic F/CaP coating process was applied on PCL scaffolds and was more effective in creating new alveolar bone, PDL, and cementum compared to uncoated scaffolds. Cell seeding with primary HPDLs cell sheets and PDLSCs on PCL scaffolds resulted in well-organized periodontal tissue complex with PDL fiber angulation similar to native tissue. On the contrary, when simple PCL scaffolds were used [12,109], PDL-like tissue was aligned parallel to the root surface, with few fibers inserting the cementum layer. A biphasic scaffold was developed, with micropatterned PLGA/PCL compartment for PDL regeneration and amorphous PCL for bone formation [130]. Each compartment was seeded with different cells, i.e., the PDL compartment with hPDLs and bone with hGFs and modified to incorporate vectors encoding BMP-7 (bone compartment) and PFGD (PDL compartment). Different combinations were evaluated in terms of either PDL compartment micropatterning or not, and gene delivery, and the optimum outcomes regarding PDL formation aligned obliquely were received for micropatterned PDL irrespectively of the single or dual gene delivery. 

Although rabbits have been used in evaluating therapeutic factors for treatment of periodontitis [146,147], their use in studies evaluating periodontal or peri-implant tissues regeneration is very limited [148,149]. This is due to their bone composition and remodeling rate which is different to human. Sowmya et al. [10] created defects in the maxilla of New Zealand White Rabbits and implanted tri-layered nanocomposite hydrogel scaffolds loaded with FGF 2 (PDL compartment) and platelet-rich plasma (PRP)-derived growth factors. They concluded that although PDL, cementum and bone was developed in the tri-layered scaffolds, more organized bone tissue was developed when the growth factors were used. 

Other animals for PDL regeneration include sheep and mice, with very limited available data. In a recent study, Vaquette et al [110] used an ovine periodontal defect model to evaluate if different cell types for seeding biphasic electrospun PCL/β-TCP scaffolds could exert a different effect on PDL regeneration and concluded that although robust cementogenesis and PDL regeneration was evidenced in cases where PDLCs and Bm-MSCs were seeded, no cementum formation was observed when GCs were used. In the study of Zheng et al. [129], β-TCP scaffolds were seeded with gene-transfected BMSCs and implanted in nude BALB/c mice periodontal defects. Cell-seeded scaffolds were able to regenerate PDL and cementum, but defects filled with neat β-TCP scaffolds presented only fibrous tissue formation without new cementum or oriented fibers. 

To summarize the findings, scaffolds alone cannot promote PDL regeneration and anchoring into new bone and cementum, irrespective of their composition or structure. In most of the studies, the use of cell seeding or loading scaffolds with growth factors was more effective in providing not only higher bone volume but also obliquely or perpendicular attachment of newly formed PDL fibers. Multiphasic scaffolds, or patterned scaffolds that mimic the structural compartments of periodontal tissues, provided the topographical cues necessary for cells to promote regeneration of PDL and the whole periodontal tissue complex. Another additional property of scaffolds towards functional PDL fibers orientation is the presence of a calcium-based component, although there is no clear evidence whether its chemical similarity to bone and cementum or its topographical orientation is the prevailing factor that guides PDL regeneration.

#### 2.2.2. Subcutaneous Placement Model

Another commonly applied model for evaluating PDL regenration involves the subcutaneous placement of scaffolds in the dorsum of athymic or nude mice and rats. Pockets of certain dimensions are surgically created on the back of rats and the materials are implanted subcutaneously. This model has the disadvantage of not resembling the actual clinical conditions in periodontal area, especially in terms of oral microflora. Subcutaneous placement of scaffolds yields results regarding the induction of any inflammatory reactions and vascularization [150]. This model is commonly applied as tissue ingrowth occurs and angiogenesis can be validated, and although timing is not the same as with human histological findings, safe predictions can be made validating the clinical translation of the model [151]. 

Regarding PDL regeneration, a few studies have used this model for pre-clinical testing of scaffolds (Supplemenatry Table S2). Biphasic or multiphasic scaffolds with PCL and β-TCP [152], or HA [153] have been tested in combination with osteoblasts, PDLCs sheets, or DPSCs. PCL/HA micropatterned 3D printed scaffolds with spatiotemporal delivery of recombinant human amelogenin, CTGF and BMP 2 from PLGA microspheres, seeded with DPSCs developed CEMP1^+^ mineralized tissue and aligned collagenous fibers resembling PDL-like tissue, while in the absence of biological cues (scaffolds without microspheres) similar tissue characteristics were received, although suboptimal [153]. Strong attachment of PDL and higher bone apposition was corelated with the CaP coating of a PCL/β-TCP scaffold seeded with PDLCs [152]. To better mimic periodontal tissue architecture in an ectopic rat model, dentin matrix or slices have been used in association to biphasic composite scaffolds [92,154,155,156]. Dentin is either treated with 37% orthophosphoric acid to expose dentin tubules [155] or treated with EDTA [92]. A patterned PDL-like layer was designed on top of dentin surfaces, with multiple perpendicularly oriented channels to guide fibroblasts alignment and increase vascularization [155]. Although this patterning resulted in vascular structures and fibers development aligned along the PDL-like layer, cementum was formed only in the case of cell seeded scaffolds. A fiber-guiding microchannel pattern from chitosan of low molecular weight with pores of 450μm and high elastic modulus was successful in guiding fibroblasts and promoting PDL regeneration [157]; however, in agreement with the study of Vaquette et al [154], PDL lacked functional orientation. Biomimetic fabrication of scaffold microarchitecture is crucial to functional orientation of new PDL tissue formation, as evidenced also by Yu et al [11], who used a bilayer scaffold of mineralized collagen and concentrated growth factor in comparison with a deproteinized bovine bone mineral. They concluded that apart from the hierarchical PDL-like microenvironment of the scaffold, its stiffness, degradation rate similar to natural bone and the good interfacial stability of the two scaffold components, allowed the smooth healing and regenerative process, leading eventually to functionally oriented PDL fibers inserted in the newly formed cementum tissue. 

Electrospun membranes have been applied to mimic the PDL and are used as an intermediate layer between dentin and the bone compartment of scaffolds [154] based on their use as suitable materials for GTR in periodontal tissues engineering. Other desirable properties of electrospun membranes are their efficacy in loading nanoparticles, antibiotics and/or growth factors. Vaquette et al [154] utilized a PCL membrane to stabilize PDL cell sheets and verified that the heat press-fitting treatment they employed improved the membrane adhesion to the scaffold and facilitated new PDL formation and attachment, although its orientation was not perpendicular to new cementum. New fibrous tissue along the dentin surfaces with large areas of no attachment were observed in mesoporous HA/chitosan scaffolds, while new cementum and continuous soft tissue formation were observed when these scaffolds were loaded with recombinant human amelogenin. 

Based on the results of the included studies a calcium phosphate mineral [92,152] or complex PDL-like patterned structures [153,155] should be present to induce the perpendicular orientation of new PDL fibers.

#### 2.2.3. Other Models

Other in vivo models include the extraction of teeth and implantation in jawbone sockets, implantation in calvaria defects and regeneration after experimental periodontitis model in maxillary molars. HA/TCP scaffolds, electrospun scaffolds or membranes from either PCL or PLGA and PEG-DA based hydrogels have been evaluated (Supplementary Table S3). Smart antibacterial hydrogels with capacity to control inflammation were used after loading with SDF-1 in an experimental periodontitis model and resulted in complete in situ periodontal regeneration with arrangement similar to normal periodontium [157]. Despite the materials used as scaffolds after implantation in jawbone defects after teeth extraction, functional regeneration of PDL was observed, along with complete regeneration of cementum and bone [72,158]. Electrospun sheets, with parallel-aligned fibers similar to ECM were efficient in allowing topographical alignment of cells to guide the development of organized PDL tissues [158]. In the model of Kim et al [159], in the case of PDL/bone removal after teeth extraction and reimplantation with aligned PCL/gelatin membranes, the authors concluded that when cells were cultured on aligned membranes under cycling mechanical loading, the combination of alignment and load was efficient in regenerating all tissues, however PDL fibers alignment deviated from normal. Nevertheless, when PDL remained in the sockets, the regenerated bone was in close contact to intact PDL without interfering connective tissues. In a rat calvaria model, Chen et al [160] evaluated electrospun multiphasic scaffolds of PCL, type I COL, and PEG-stabilized ACP nanoparticles loaded with rhCEMP. They reported cementum-like tissue formation, limited bone regeneration, and thick connective tissue formation with parallel oriented fibers. 

### 2.3. Clinical Studies with Scaffolds for PDL Regeneration

#### 2.3.1. Clinical Studies Involving Scaffolds and Growth Factors

The regenerative therapies in treatment of periodontitis implicate various bone grafts and GTR aimed to promote de novo formation of periodontal complex (Table 3). Although much effort was made in development of new scaffolds for periodontal tissues regeneration at preclinical level, there are limited data on their clinical application. 

Safety and efficacy of natural and synthetic scaffold materials currently used in clinical periodontology are well documented. Nevertheless, a new synthetic zinc-substituted nanostructured material based on monetite (Sil-Oss^®^) for the treatment of intra-bony defects was developed and tested in 30 patients [176]. The authors did not find significant differences with synthetic HA in terms of clinical findings and bone mineralization; however, the new material showed a significant increase in bone fill percentage as compared with HA at 3 and 6 months of observation.

With development of CBCT and 3D printing technologies it became possible to construct patient specific scaffolds. 3D printing provides better control over the scaffold microarchitecture and allows fabrication of complex multistructures, recreating features of bone, cementum, and PDL. In 2015, for the first time Rasperini et al. [169] fabricated custom-made PCL scaffold using selective laser sintering and implanted it into a large periodontal defect of a 53-year-old male. The scaffold contained an internal compartment for rhPDGF-BB delivery and extended pegs for PL regeneration and guidance. After one year of follow there were no signs of chronical inflammation and the scaffold remained covered, favoring partial root coverage, and 3 mm of clinical attachment gain. However, it later became exposed to the intraoral environment, contaminated by microbes and, consequently, lost. Apparently, the slow degradation time of PCL polymer and mismatch of its mechanical properties with the surrounding tissues was the reason for its failure. Another clinical case of horizontal alveolar bone augmentation by 3D printed bioceramic (30% HA-70% β-TCP) scaffold was recently reported by Mangano et al. [181]. The authors demonstrated histological and histomorphological assessment of this retrieved scaffold after seven years of implantation. Interestingly, despite complete integration of biomaterial, which remained unloaded for so many years, it was not fully resorbed and even preserved its initial microarchitecture. 

In recent years, the biological growth factors have been extensively applied for periodontal regeneration as components of biomimetic scaffolds or locally in a form of solution. Local delivery of bioactive molecules by scaffolds can create favorable microenvironment for differentiation of stem cells in the surrounding periodontal tissues. The most frequently used delivery system for growth factors is tricalcium phosphate (β-TCP). Due to its porous microstructrure it entraps biological factors, helps in stabilization of blood clot, and serves as a scaffold for new bone formation. 

Several case series and randomized controlled clinical trials utilize rhPDGFBB [165,166,167], enamel matrix protein derivative (EMD) [182], fibroblast growth factor-2 [171,172,178], and cell binding peptide (P-15) [164] to stimulate regeneration of periodontal bone defects and soft tissues. 

PDGF was discovered in 1989 by Lynch and co-workers and since then is thoroughly investigated as a treatment option for regeneration of the whole periodontal complex. PDGF can bind the superficial receptors of periodontal ligament cells and bone cells and enhance their chemotaxis and proliferation [184,185]. It can also promote angiogenesis and wound healing by stimulation of VEGF release [186]. Sarment et al. [162] found that locally delivered rhPDGF stimulates release of pyridinoline cross-linked carboxyterminal telopeptide of type I collagen and enhancing bone turnover.

Nevins et al. [167] demonstrated encouraging long-term results of rhPDGF application for treatment of large osseous defects. During the observation period of 36 months, they noticed significant clinical and radiographic improvements in patients that were treated by rhPDGF-BB at 0.3 mg/mL with TCP as compared to other treatment modalities. The authors defined the positive treatment outcome when clinical attachment level inceased by 2.7 mm and linear bone growth was higher than 1.1 mm.

Safety and efficacy of rhPDGF was documented by Jayacumar et al [166]. They found significantly higher linear bone growth (3.7 mm vs. 2.8 mm) and bone fill (65.6% vs. 47.5%) in the experimental group as compared to the TCP control after 6 months over baseline, while no pronounced adverse effects such as pain, fever or swelling were noticed. Similar results were reported by Maroo and Murphy in 2014 [168], who have found even greater gain of the amount (4.05 mm vs. 2.50 mm) and percentage of defect fill (94.3% vs. 68%) at the rhPDGF treated sites compared to TCP after 9 months of follow up.

Another application of rhPDGF for management of gum recession has been reported by McGuire and Scheyer [165]. In this case series, it was suggested that application of TCP, rhPDGF, and collagen membrane is similarly effective for the reduction of gingival recession as subepithelial connective tissue graft. Three years later, a randomized clinical trial of this group demonstrated the regeneration of the periodontal complex when using rhPDGF-mediated therapy through histological analysis. Particularly, in 9 months post surgery, the rhPDGF-treated sites showed oblique orientation of the Sharpey’s fibers and their insertion into newly formed cementum [165]. 

Several clinical trials utilize FGF-2 in combination with resorbable scaffolds for periodontal defect treatment [171,172,178]. FGF-2 stimulates angiogenic and mitogenic activity of periodontal ligament MSCs and plays an important role in wound healing. Randomized clinical trials showed that administration of 0.3% of rhFGF-2 significantly improved the bone fill percentage, and its superior efficacy compared to EMD treatments [171,172]. Periodontal therapy with FGF-2 was found to be efficient in smokers. 

Platelet-rich plasma (PRP) and platelet-rich fibrin (PRF) contain a cocktail of growth factors such as PDGF, VEGF, insulin-like growth factor, and transforming growth factor which accelerate wound healing and new bone formation. Recent meta-analysis pointed out favorable clinical outcomes for treatment of periodontal intarabony defects using PRF in combination with bone graft materials [187]. However, yet there is no histological evidence in clinical studies that blood plasma coagulation can promote true periodontal regeneration. 

#### 2.3.2. Clinical Studies Involving Caffolds Combined with Cells

Another promising approach for periodontal tissue regeneration is stem cell-based therapies There are several sources of stem cells that were described in literature such as bone marrow, PDL and dental pulp, exfoliated deciduous teeth, gingival and human umbilical cord. Isolation of allogenic MSCs from human umbilical cord is not invasive and is less expensive than the convetional cell isolation procedures. Besides, these cells are pluripotent, as they can differentiate into osteoblasts, cemetoblasts and PL fibroblasts and are capable for self-renewing. In this direction, Dhote et al. [188] applied umbical cord MSCs in combination with β-TCP scaffold and platelet-derived growth factor-BB (PDGF-BB) and found significant gain of clinical attachment level and radiographic defect fill as compared to open flap debridement (OFD). Ferrarotti et al [189] used autologous DPSC micrografts collected and directly seeded on collagen sponges for treatment of chronic advanced periodontitis. The authors reported improved clinical and radiographic parameters as compared to control sites treated with collagen sponges alone. Safety and favorable clinical results were reported also by Baba et al. [190], who treated inrtabony defects in 10 patients with autologous bone marrow stem cells in complex with PRP in a woven-fabric composite poly-L-lactic acid scaffold. However, there was no control group for further comparisons. By contrast, Chen et al. [191] demonstrated no significant differences in clinical and radiographic findings between demineralized bovine bone scaffolds with PDL-derived MSCs compared to the scaffold alone. During 12 months of follow up, the clinicians did not observe considerable adverse effects and changes in blood formula related to MSCs and scaffolds implantation and considered that it was safe. Sanchez et al. [192] performed a pilot clinical study on 20 patients and did not find additional clinical benefits of using PDL MSC-based cell therapy as compared to xenogenic bone substitute alone after 12 months.

In a more recent study by Apatzidou et al. [193], three options of periodontal bone defects treatment were compared: a complex of alveolar bone marrow MSCs with collagen scaffolds and autologous fibrin/platelet lysate (aFPL), a collagen scaffold with aFPL and no scaffold as a control. Although there were no inter-group differences after 12 months of follow up, all treatment approaches led to significant clinical improvements in terms of radiographical bone fill and soft tissue healing. The authors suggested that application of MSCs based therapy might have potential in defects with a complicated, non-contained morphology over the extended period. 

**Figure 3:**
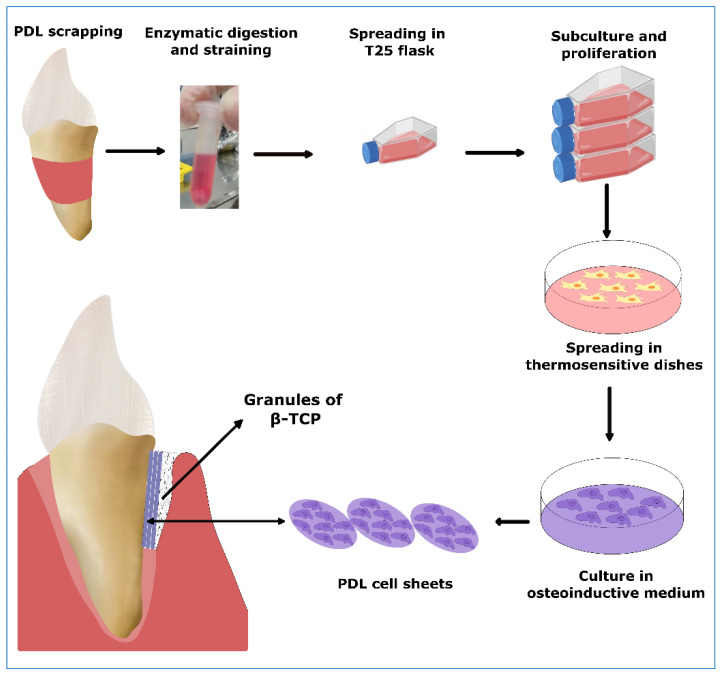
Schematic representation of the clinical model applied by Iwata et al. [70] for cell sheet transplantation.

Quite a different approach for severe bone defect treatment was reported by Iwata et al. [8], who applied PDL-derived cell sheets in combination with TCP (Figure 3). Clinical and radiographic findings after 6 months revealed clinical attachment gain (2.5 ± 2.6 mm) and increase of bone height of 2.3 ± 1.8 mm. The results maintained over the follow up period, while no serious adverse effects were recorded.

Several clinical studies utilize autologous GFs seeded on bioresorbable scaffolds such as collagen [194,195,196], plasma mesh rich in growth factors [197] and acellular dermal matrix allograft (ADMA) [198] for management of gingival recession. This cell tissue engineering strategy does not require extensive grafting, as a very small piece of gingiva is collected, so it decreases patients’ morbidity. Favorable results of GF-based therapy on collagenous matrix in terms of recession coverage and increase of keratinized tissue width were reported by Mohammadi et al. [195] and Dominiak et al. [194] at 3 and 6 months, respectively. Histological evaluation revealed complete resorption of collagenous membrane in 3 months, formation of new keratinized tissue and improvements in tissue healing in the experimental group. In a randomized controlled clinical trial, Jhaveri et al. [198] compared human autologous fibroblasts seeded on acellular dermal matrix graft with a combination of a connective tissue graft (CTG) and coronally advanced flap (CAF) and did not observe statistical differences in clinical measurements among groups. Milinkovich et al. [196] also found conventional CTG technique more effective for gingival recession management in terms of keratinized gingiva width than the experimental GF-rich collagenous scaffold during a 12-month observation period. 

From the clinical studies involving cells and scaffolds for periodontal tissue regeneration (Table 4) it can be summarized that the efficiency of MSCs-based therapies for periodontal regeneration is questionable and should be further evaluated in histological and long term randomized clinical trials. Apart from that, several important issues such as safety, immunogenicity of MSCs, potential risks and cost-efficiency should be considered before the implementation of cell-based periodontal therapies as a treatment option in clinical practice.

## 3. Discussion and Concluding Remarks

Different materials have been used for the development of scaffolds for PDL regeneration and evaluated in vivo. Among them aliphatic polyesters such as PLA, PGA, their copolymer PLGA, and PCL have been extensively investigated [155]. PCL in particular is the most commonly used polymer, either applied in the form of membranes or in composite scaffolds, with other polymers such as PLGA or inorganic minerals such as HA or TCP. This preferable use of PCL derives from its availability, relatively low cost, and high modification potential [156]. Although it is a highly crystallized material (50–60%) with slow hydrolysis in vivo, its physicochemical and mechanical properties are easily tailored to meet different needs. Electrospun PCL membranes have been developed for combined drug delivery [9] and as barriers to cover periodontal defects [109], electrospun or printed PCL scaffolds, alone [12,132], combined with β-TCP [110,154] or HA [153] to provoke hard tissue formation and electrospun PCL/PLGA [123,130] or multiphasic PCL/COL/PEG scaffolds [160] for better mimicking periodontal tissue architecture and cementum formation. Calcium phosphate minerals or biomimetically developed calcium phosphate layers have been used in various forms to promote periodontal regeneration. Among them HA [13,72,92,123,126] and β-TCP [62,72,92,124,129,201] are the most popular, followed by bioactive glasses [10,121] and other ceramics [116]. Calcium phosphate coatings were applied in two in vivo studies [12,152] that yielded higher bone or cementum apposition. In clinical applications, β-TCP is the material used almost exclusively, despite that when not-combined with other scaffolding materials or growth factors does not seem to effectively regenerate periodontal tissues [163,165,166,167]. Another calcium phosphate scaffold was developed by 3D-printing from biphasic calcium phosphate (BCP) and applied in one case study [181], while BCP was also used in **a** recent randomized clinical study [183]. This composite material consisting of HA and β-TCP at a proportion of HA to **β**-TCP of 60:40, provides a balance over the high degradation rate of β-TCP and the low dissolution of HA, in an effort to mimick bone resoprption rate [202]. Scaffolds in the form of hydrogels and sponges have also been used for periodontal regeneration [115,119,131,133,157,189,199]. In this respect, collagen is the predominant material [115,119,195] used combined with HA [123], FGF2 [125], cell binding peptide (P-15) [164] and BCP [183]. Collagen hydrogels have been used effectively as drug carriers and possesses significant properties, such as feasible synthesis, affordable cost, low toxicity, and ease of use [203]. Other hydrogel scaffolds [203] such as chitosan-based [131], gelatin [133], PEG-DA and DTT [157], and self-assembling peptide hydrogels [128] have also been used in in vivo studies.

None of the materials used in scaffolds for periodontal regeneration has profound advantages over the others and in most situations the different materials combinations produce better results. The construction of multi phasic scaffolds with different compartments of biomimetic microtopography and patterning seems to be the optimum choice for scaffolds manufacture, as it successfully guides cells to develop fibrous PDL-like tissues with the appropriate orientation. Despite the enhancements of manufacturing technology for complex scaffold constructs, their morphology still is far away the normal architecture of periodontal tissues. The employment of new scanning systems that can transfer with high accuracy the actual dimensions of a periodontal defect to contemporary milling machines that can use sophisticated technology to produce desirable scaffold compartmentalization and surface micro- or nanopatterning and topolography can lay the foundation of personalized treatment of degenerated periodontal tissues. However, this approach has inherent drawbacks such as cost and limited resolution to accommodate the complicated nanotopography of the periodontal architecture.

Animal models have proven considerably important for evaluating different materials and approaches to regenerate periodontal tissues. However, they are unable to mimic clinical conditions as in most cases the defects are surgically created, not resembling the actual destruction sites after periodontitis establishment, and they can not include contributing factors such as the presence of aggressive bacteria species, systemic diseases, smoking, occlusal parafunction, or host response. Another point in evaluating scaffolds for PDL regeneration in models that do not include the in situ periodontal tissue environment, like subcutaneous placement or calvaria models, is the absence of clinically relevant mechanical loading that could affect the regeneration response by modulating the cellular and molecular pathways needed. Although the established protocols are more or less commonly applied in most of the studies, still there is a lot of heterogeneity among study methodologies and materials applied, that makes almost impossible the direct comparison of findings to draw safe conclusions. 

Periodontal ligament regeneration requires the simulataneous regeneration of bone and cementum. Although new bone formation has been observed in most of the studies, cementum regeneration remains a challenging task in vivo, as the identification of expression markers and differentiation pathways has not been specific. Some of the markers that have been determined so far for the identification of cementoblasts are the cementum attachment protein (CAP) and the cementum protein-1 (CEMP-1) [204,205]. CEMP-1 has been identified in cementoblasts and its progenitor cells, overexpression of which in PDLCs promotes cementoblastic differentiation, while reduced expression indicates osteoblastic or periodontal differentiation [206]. During the early stages of cementogenesis, an increased expression of miR-628-5p, SPON1, and PTPLA has been observed, while CEMP-1 expression is restricted by the presence of miR-628-5p and miR-383 [207]. CEMP-1 and PDPLA expression is increased in the late stages of cementogenesis [207]. 

Cell sheet engineering has been proven an effective treatment strategy regarding the regeneration of the full periodontal complex, consisting of alveolar bone, cementum, and PDL tissue, mainly in preclinical models, but also in one clinical study. PDLSCs emerge as the most suitable cell source for periodontal cell sheet engineering. Cell sheet engineering application face challenges related to cell therapies, in general, as well as the specific technique. Cell isolation and in vitro expansion are costly procedures that must be controlled by specific regulations, thus limiting the availability of such therapies to the general population. Additionally, cell sheet engineering is a technically demanding procedure, where handling and stabilizing the construct can be challenging, while proper attachment in situ is crucial for the desired regenerative outcomes [58]. Autologous transplantation of PDLSC sheets has been proven effective and safe in preclinical studies, as well as in a clinical study. However, there are often limitations that hinder this therapeutic strategy, such as lack of cell source, presence of periodontitis, and patient’s age [208]. As a procedure, cell isolation and expansion towards the fabrication of a consistent and stable product following quality standards with Good Manufacturing Practice (GMP) can be a time-consuming and expensive process, thus it does not always present as a favorable or attractive treatment choice for the patient [208]. Changing direction towards allogeneic cell source in order to create cell banks and readily available products, could be more time- and cost-efficient, while also providing a possible treatment for patients with the above-mentioned limitations. Application of allogeneic cell sheets have been proven a safe and effective alternative [104,106].

PDL regeneration on Ti surfaces with the application of cell sheet engineering has been proven feasible, and it is thought to be beneficial regarding the long-term survival of dental implants, through the prevention of bacterial invasion and protection against inflammatory peri-implantitis [68], and through the dispersion of increased occlusal load. Nonetheless, those assumptions have not been supported by clinical data yet, and the long-term stability of the interface between Ti surface and the newly regenerated PDL-like tissue needs further assessment. 

The approach of tissue regeneration based on combination of scaffold material, stem cells MSCs, and/or bioactive molecules might be an alternative for conventional periodontal surgical treatments in clinical practice. Due to the divergence of treatment outcomes in reported clinical trials and clinical case series, the benefits of scafold-based therapies are ambiguous. Most of clinical trials are focused on alveolar bone regeneration and evaluate clinical parameters such as clinical attachment level, and radiographical bone defect fill. There are limited data about histological analysis to confirm periodontal regeneration in humans due to ethical isuues. Further assessment of risk benefit and adverse effects should be performed in large-cohort studies.

## Figures and Tables

**Table 1:** In vivo studies employed orthotopic models to assess the regenerative capacity of cell sheet transplantation in periodontal defect models, with or without biomaterials.

Author	Cells	Experimental Groups	Additional Pretreatment	Material	Technique	Experimental Setup	Results
Nakajima et al., 2008 [99]	HGFs	FN-ALP sheet group, FN sheet group, control (no treatment in the defect), control (without immunosuppressant FK administration)	None	None	FN matrix-based multilayered cell sheets of hGFs modified to express ALP (FN-ALP)	Orthotopic model of fenestration bone defects in rats	FN- ALP-expressing hGFs supported the regeneration of cementum-like, PDL-like and bone tissue, exhibiting superior regenerative potential.
Ding et al., 2010 [100]	minipig PDLSCs	Control group, HA/TCP group, HA/TCP scaffolds +autologous pPDLSCs group, HA/TCP scaffolds + allogeneic Guizhou minipig pPDLSCs group, HA/TCP scaffolds+ autologous heterogenic minipig pPDLCs group	None	HA/TCP	Cell sheet	Orthotopic model of experimental periodontitis in minipigs	Treatment containing either autologous or allogeneic pPDLSCs resulted in PDL-like tissue regeneration. The use of allogeneic cells did not result in immunological rejection.
Tsumanuma et al., 2011 [101]	Canine PDLSCs, BMMSCS, and APCs	Control, PDLC group, BMMSC group, APC group	None	Woven PGA, porous β-TCP and 3% type I collagen	Three-layered cell sheets attached with PGA	One-wall defects were surgically created in dog	The PDLC group exhibited enhanced cementum-like and PDL-like tissue regeneration, exhibiting more dense collagen fibers and thicker mineralized tissue.
Wei et al., 2012 [75]	PDLSCs	Vc-induced autologous PDLSCs sheet group, UpCell dish PDLSCs sheet group, Gelfoam scaffolds/dissociated autologous PDLSCs group (control)	Vc treatment	Gelfoam scaffold	Cell sheet	Ectopic transplantation in nude mice, and orthotopic transplantation experimental periodontal lesions bone defect in miniature swines	Vc-induced PDLSCs sheet group and UpCell dish PDLSC sheet group application resulted in significantly more bone/cementum-like tissue formation compared to control/Vc-induced PDLSCs sheet group was significantly better.
Zhao et al., 2013 [102]	PDLSCs	PDLSCs/PRF construct group, cell sheet fragments group, PRF granules group	None	PRF granules	Combination of fragments from PDLSCs cell sheet and PRF granules	PDLSCs/PRF granules construct in tooth reimplantation in dogs	PDLSCs/PRF construct promoted PDL-like tissue regeneration and exhibited reduction in terms of inflammation and ankylosis.
Iwasaki et al., 2014 [103]	PDLSCs	Amnion group, PDLSC-amnion group	None	Decellularized amniotic membrane (amnion)	None	Application of PDLSCs -amnion in a periodontal defect model in rat maxillary molars	Histological and radiographic analysis showed that PDLSC-amnion group promoted PDL-like tissue regeneration.
Guo et al., 2014 [86]	Rat PDLSCs	MCPs group, MUCPs group, MCPs/TDM group, MUCPs/TDM group	None	TDM	MCP and MUCPs produced by MCS and MUCS	In vivo transplantation of MCPs or MUCPs into the imental pouch; periodontal defect model and in vivo transplantation of MCPs and MUCPs in rats	All groups promoted cementum-like and PDL-like tissue regeneration, but MUCPs group exhibited superior behavior in terms of mineralization and collagen fiber arrangement compared to MCPs group.
Cao et al., 2015 [104]	hDPSCs	Control group, hDPSC injection group, HGF-hDPSC injection group, hDPSC sheet, HGFhDPSC sheets	Adenovirus-mediated transfer of HGF gene to DPSCs	None	Cell sheet of adenovirus-mediated transfer of HGF	40 periodontitis lesions, three-wall intrabony defects, in the 1st molars of miniature pigs	HGFhDPSC sheet group was able to promote PDL-like tissue formation and alveolar bone regeneration similar to that of native tissue, whereas the other groups provided only limited regeneration.
Hu et al., 2016 [105]	hDPSCs	Control group, hDPSC injection group, hDPSC sheep group	None	None	Cell injection or cell sheet transplantation	Three-wall intrabony periodontal defects, in miniature pigs	Both experimental groups effectively promoted periodontal regeneration compared to control. hDPSC sheet application resulted in significantly better bone regeneration compared to the hDPSC injection.
Tsumanuma et al., 2016 [106]	Canine PDLSCs	Control group, autologous group, allogeneic group	None	Woven PGA, porous β-TCP and 3% type I collagen	Three-layered cell sheets attached with PGA	Critical size supraalveolar periodontal defect model in dog	Both autologous and allogeneic groups were able to regenerate bone, cementum-like and PDL-like tissue.
Yu et al., 2016 [90]	PDLSCs	Inflammation group, hypoxia group, inflammatory plus hypoxic stimuli-dual-stimuli group, no-stimulus group, blank group, CBB group	Inflammatory conditions (inflammation), hypoxic conditions (hypoxia), or a combination of both (dual stimuli) conditions	CBB	Cell sheet	Ectopic trasplantation model (subcutaneously) into the dorsal region, and orthotopic model with surgical creation of periodontal defects (3 mm × 1.5 mm) in nude mice	Hypoxia group exhibited more bone formation compared to other groups, while cementum-like and PDL-like tissue formation was identified in the no-stimulus and hypoxia groups.
Guo et al., 2017 [107]	PDLCs and DFCs	Control group, DFC sheet group, PDLC sheet group	*P. gingivalis* LPS-induced inflammation microenvironment	None	Cell sheet	Canine periodontitis model (two wall intrabony defects), in dogs	DFC sheet application was more effective in terms of bone, cementum-like and PDL-like tissue regeneration compared to the PDLC sheet.
Takewaki et al., 2017 [108]	BMMSCs	No graft, C-MSC in growth medium, C-MSC in OIM	Osteoinductive medium (OIM)	None	MSC/ECM complex (C-MSC)	Orthotopic model of class III furcation defect, in beagle dogs	Both C-MSC and C-MSC-OIM exhibited formation of cementum-like, PDL-like and bone formation leading to the regeneration of the periodontal complex.
Farag et al., 2018 [109]	PDLCs	Scaffold group, decellularized cell sheet/scaffold group	None	PCL	Decellularized cell sheet	Rat periodontal defect model in the mandible (orthotopic)	PDL-like tissue regeneration was observed in both groups. However, the group with the decellularized cell sheet presented higher detection of PDL fiber attachment with perpendicular orientation.
Iwata et al., 2018 [8]	PDLCs	PDL cell sheet group	None	β-TCP granules	cell sheet	Bony defects were filled with three-layered PDL-derived cell sheets and with β-TCP granules (clinical study)	Improvement was observed in terms of bone regeneration and clinical attachment 6 months after application of PDL-cell sheet.
Yanget al., 2019 [94]	DFCs and SHEDs	SHEDSs combined with TDM group, DFCSs combined with TDM group, TDM group	None	TDM	Cell sheet	Subcutaneous transplantation into nude mice and orthotopic implantation in Sprague-Dawley rats’ jawbone	Both SHEDs/TDM and DFCs/TDM groups formed PDL-like tissues, enriched in collagen fibers and fibroblasts, with arrangement similar to that of native PDL.
Vaquette et al., 2019 [110]	GC, BMMSCs, and PDLCs	Control group (no cells on scaffold), GC group, BMMSC group, and PDLC group	None	PCL	Biphasic PCL scaffold consisting of bone and PDL compartments combined with the cell sheets.	Dehiscence periodontal defects in sheep	Bone, cementum-like and PDL-like tissue regeneration was observed in the BMMSC and PDLC groups compared to the GC and control group.
Yang et al., 2019 [111]	hDFCs	Blank group, cDFCSs group, TDMP group, HA/-TCPgroup, TDMP + cDFCSs group, HA/-TCP + cDFCSs group	None	TDM particles or HA/-TCP	Cell sheet	One-wall periodontal intrabony defects in beagle dogs	The use of materials enhanced bone formation. The presence of DFCs promoted the regeneration of bone and PDL-like tissue.
Raju et al., 2020 [97]	Rat PDL cells and osteoblastic cells	PDL cell sheet group, MC3T3-E1 cell sheet group, complex cell sheet group (containing both cells)	None	None	Cell sheet	Ectopic and orthotopic transplantation in vivo in mice	Ectopic transplantation of complex cell sheet resulted in PDL-like and bone tissue formation. Only complex cell sheet group was able to regenerate bone and PDL-like tissue similar to the native PDL-bone complex.
Jiang et al., 2021 [112]	hPDLCs	dHPDLC group, dHPDLC sheets loaded with PCL/GE group (dHPDLC-PCL/GE), control group	None	PCL/GE nanofibers and 15-deoxy-Δ12,14-prostaglandin J2 (15d-PGJ2) nanoparticles	Decellularized cell sheet	Periodontal defects (periodontal fenestration defect) in rats	dHPDLC and dHPDLC-PCL/GE groups promoted new bone formation as well as PDL-like and cementum-like tissue regeneration compared to control. dHPDLC-PCL/GE group exhibited irregular and perpendicular fiber orientation in the regenerated PDL-like tissue.

**Table 2:** In vivo studies evaluating scaffolds for PDL regeneration employing the periodontal defect model.

Study	Scaffold Type	Cells	In Vivo Animal Model	Animal/ Evaluation Time	Major Findings
Zhang et al., 2009 [113]	Hybrid tooth constructs from PGA/PLLA and PLGA scaffolds for tooth and bone parts respectively	DSCs	Intrabony defects in the mandible	Yucatan mini pigs 12 and 20 weeks	Cementum-like tissues but absence of periodontal ligament tissues. Scarcely found fibers resembling Sharpey’s fibers penetrated the regenerated cementum-like tissues and surrounding bone
Park 2012 et al. [114]	Amorphous and fiber guiding PCL scaffolds	hPDLs	Periodontal defect model with osseous defects on the buccal side of the mandible	Athymic rats 4 weeks	Cementum-like tissue was formed on the dentin surfaces with fiber guiding scaffolds, which displayed similar angulation of fiber orientation to native ligament tissue
Inukai 2013 et al. [115]	Absorbable collagen sponges	dMSCs and dPDLCs	One-wall intrabony defects on 2nd, and 4th premolars	Hybrid dogs 4 weeks	New lamellar and woven bone formation and cementum with dense collagen fibers in the MSCs condition medium+ scaffold group
Wu 2013 et al. [116]	Porous nagelschmidtite (NAGEL: Ca_7_P_2_Si_2_O_16_) bioceramic and β-TCP scaffolds	No cells	Periodontal defect model, defects on 2nd and 3rd maxillary premolars and 1st maxillary molar	Beagle dogs 4 and 8 weeks	Both materials presented new bone, cementum and PDL tissue formation, but thicker osteogenic layer was observed for the NAGEL group compared to β-TCP
Chantarawaratit 2014 et al. [117]	Acemannan sponges	No cells	Class II furcation defects of maxillary and mandibular 2nd and 3rd premolars	Mondrel dogs 30 and 60 days	New bone, cementum and PDL formation at 30 d. Accelerated regeneration for the acemannan treated groups
Fawzy El-Sayed 2015 et al. [118]	IL-1-receptor-antagonist (IL-1ra) releasing hyaluronic acid synthetic extracellular matrix (HA-sECM).	G-MSCs	Periodontal defect model with defects on premolars/molars	Miniature pigs 16 weeks	Cementum-like substance, bone and PDL were regenerated in the IL-1ra/G-MSCs/HA-sECM, and G-MSCs/HA-sECM groups and Sharpey’s fibers similar to normal periodontal tissues
Kato 2015 et al. [119]	Collagen hydrogel scaffold (Col)	No cells	One wall intrabony defects on mandibular 2nd and 4th premolars	Beagle dogs 4 weeks	Scaffolds with rh-BMP-2: considerable new trabecular alveolar bone, thick, cellular cementum like tissue with Sharpey’s fiber insertion. Fiber-rich PDL
Jiang 2015 et al. [120]	Three-dimensional multilayered scaffold (3D): aligned (AL) and random (RD) biodegradable PCL-PEG (PCE) copolymer electrospun nanofibrous mats into porous chitosan (CHI)	No cells	Periodontal defect model with fenestration defects on maxillary 1st molars	Sprague–Dawley rats 8 weeks	strong topographical guidance of scaffolds to the PDL regeneration. 3D-RD and 3D-ALscaffolds lead to the regeneration of tissues with mostly defined orientation, while 3D-AL scaffolds resulted in cementum-like tissue formation on dentin surfaces.
Zhang 2015 et al. [121]	MesoPorous BioGlass/silk scaffold containing adPDGF-B and adBMP7	No cells	Periodontal defect model with defects on 2nd and 3rd maxillary premolars	Beagle dogs 8 weeks	Best results with the adPDGF-B+ BMP scaffolds” PDL regeneration at 90% of its original height along with both alveolar bone and cementum formation with multiple new Sharpey’s fibers
Cai 2015 et al. [122]	PLGA-PCL scaffold by electrospinning	BMSCs cultured in multilineage differentiation (FGF-2), osteogenic (O^+^) and chondrogenic (C^+^) medium	Periodontal defect model with intrabony three-wall defects on maxillary 1st molars	Fischer rats 6 weeks	Newbone and ligament, cementum formation limited to the apical root surface. Collagen fibers with an oblique orientation in the FGF-2 and C^+^ groups, while cartilage-like tissue formation in the C^+^ group. Bone formation was more profound but limited collagen fibres were observed in the O^+^ group
Liu 2016 et al. [123]	Collagen-hydroxyapatite scaffold (CH)	BMSCs	Labial alveolar intrabony defects in 2nd premolars	Beagle dogs 12 and 24 weeks	Newly formed alveolar bone, PDL and cementum after 12 weeks and after 24 weeks mineralized bone and well-organized and defined tissues
Ogawa 2016 et al. [124]	Nano β-TCP and FGF-2-loaded nano-β-TCP scaffold, collagen scaffold as control	No cells	One wall intrabony defects on mandibular 2nd and 4th premolars	Beagle dogs 10 days and 4 weeks	FGF2-treated scaffold: acellular cementum-like tissue in continuity with pre-existing root cementum and PDL-like tissue
Momose 2016 et al. [125]	Collagen Hydrogel Scaffold and FGF2	No cells	Artificial buccal class II furcation defects on the mandibular 2nd, 3rd and 4th premolars	Beagle dogs 10 days and 4 weeks	New bone and vessel-like structures in the FGF2-loaded scaffolds. Formation of woven bone. Only fibrous tissue on the root surface but not PDL attachment. Inhibition of epithelial tissue infiltration
Gonçalves 2016 et al. [126]	PisPLLA and PLLA, PLLA-30% HA, PLLA-COL-30% HA, PLLA-COL-30% HA-BMP7 membranes	SHEDs	Periodontal defect model with fenestration defects on 1st mandibular molars	Wistar rats 4 weeks	Both PLLA/COL/HA and PisPLLA/COL/HA membranes presented high bone and PDL regeneration, but the PLLA/COL/HA presented thicker cellular cementum and remained intact for the testing period. The presence of cells inhibited bone regeneration.
Zang 2016 et al. [127]	Chitosan/anorganic bovine bone (C/ABB) scaffolds	hJBMMSCs	One-wall intrabony defects on 3rd premolars and 1st molars	Beagle dogs 8 weeks	Bone and cementum formation were greater in groups C/ABB and C/ABB+cell, with the later presenting more lamellar bone and dense PDL with oblique or perpendicular embedding in the new formed tissues
Takeuchi 2016 et al. [128]	Self-assembling peptide hydrogel (RADA16))	No cells	Periodontal defect model with bilateral defects on 2nd maxillary molars	Wistar rats 4 weeks	Ambudant new bone formation was observed. PDL-like collagen bundles with oblique orientation to root surface
Zheng 2017 et al. [129]	β-TCP scaffolds	Ad-hLEP-EGFP and Ad-EGFP transfected BMSCs	Periodontal defect model with defects on 1st and 2nd molars	Nude BALB/c mice 10 days and 4 weeks	New well-organized PDL fibersincerting cementum-like tissue for the cell seeded scaffolds. Cementum generation was more pronounced at the β-TCP scaffolds +Ad-hLEP-EGFP-transfected BMSCs group and completely absent in the β-TCP group
Sowmya 2017 et al. [10]	Tri-layered scaffold: a. Cementum: CHI- PLGA)/nBGC/ CEMP1, b. PDL: CHI–PLGA/FGF 2, and c. Bone: CHI–PLGA/nBGC/PRP	No cells	Periodontal defect model with maxillary defects	New Zealand white rabbits 4 and 12 weeks	More formation of new cementum, fibrous PDL, and alveolar bone with well-defined bony trabeculae for scaffolds with growth factors
Pilipchuk 2018 et al. [130]	Biphasic scaffods. PDL structure: Micropatterned, PLGA/PCL (AdPDGF-BB) Bone: amorphous PCL (AdBMP-7).	Scaffolds’ bone region was seeded with hGFs and the PDL region with hPDLs	Periodontal defect mode with fenestration defects on 1st mandibular molars	Athymic rats 3 and 6 weeks	Soft tissue for all groups by 3 weeks obliquely aligned in the patterned scaffolds. New soft tissue was more mature and PDL-like tissue for the groups with combined patterning and gene delivery.
Chien 2018 et al. [131]	Injectable and thermosensitive chitosan/gelatin/glycerol phosphate hydrogel	IPCs, loading with BMP-6	Periodontal defect model with defects on maxillary 1st molars	Sprague Dawley rats 4 weeks	Only the iPSCs-BMP-6-hydrogel group showed new bone, cementum and PDL formation
Farag 2018 et al. [109]	PCL melt electrospun scaffolds and electrospun PCL sheet as barrier to cover the periodontal defect	Primary hPDLCs decellularized cell sheet that enveloped the scaffold	Periodontal defect model with intrabony defects in the mandible	Athymic rats 2 and 4 weeks	PCL scaffolds: fiber orientation parallel to the root surface with few isolated areas of inserted fibers into the cementum surface. Decellularized scaffold constructs: organized fibers mostly inserted perpendicularly to the tooth surface
Vaquette 2019 et al. [110]	Biphasic electrospun PCL scaffold + β-TCP 20% wt	Scaffolds seeded or not with PDLCs, GCs, and BMMSCs	Periodontal defect model with defects adjacent to the 2nd pre-molar and 1st molar of the mandible	Sheeps 5 and 10 weeks	Newly formed cementum and bone, oblique PDL fiber insertion and periodontal regeneration with vascularized PDL significantly higher in the PDLCs and BMMSCs
Yang 2019 et al. [132]	2D PCL nanofibers and 3D PCL nanofibrous (aligned or random) scaffolds	PDLSCs	Periodontal defect model with fenestration defects in mandibular buccal sides	Sprague-Dawley rats 3 and 6 weeks	PDL-like thick collagenous tissue, well aligned and inserted into the newly formed bone for the aligned 3D scaffolds
He 2019 et al. [133]	Transglutaminase crosslinked gelatin hydrogel (TG-gel)	No cells Interleukin IL-4 stromal cell-derived factor SDF-1a	Periodontal defect model with defects on 2nd molars	Sprague-Dawley rats 1, 4, and 8 weeks	Newly formed and oriented PDL, new bone and new cementum in all hydrogel groups. The presence of two cytokines provided the best outcome
Wang 2020 et al. [134]	nHA/BFGF composite scaffold	No cells Geistlich bio-Gide (GBG) membrane	Periodontal defect model with defects in root bifurcation area of premolars	Dogs 6 weeks	More new bone, cementum and PDL formation for the nHAC/BFGF/GBG implantation group
Huang 2020 et al. [13]	Biphasic scaffold: gelatin and β-TCP/HA particles (BH) and biphasic cryogel scaffold (BCS)	No cells. BMP-2 infusion in scaffolds and EMD on root surface prior to implantation	Periodontal defect model with two-walled intrabony defects on mandibular 2nd and 4th premolars	Beagle dogs 12 weeks	Cementum with interposing Obliquely inserted ligament-like fibers to the newly formed bone. The functionally graded membrane provided additional limited benefit
Ding 2020 et al. [135]	Composite PLLA-PLGA fibrous scaffolds through coaxial electrospinning of core and shell solutions	No cells	Intrabony bone defects distal to the front of the mandible, 1 mm apical to the alveolar bone crest	Wistar rats 1, 2, 4, and 8 weeks	New PDL formation with similar angulation with the natural PDL and in general in situ cementum–ligament–bone complex regeneration with the growth factors (bFGF and BMP-2) loaded scaffolds
Shang 2021 et al. [136]	PLGA fibrous membranes incorporating DMOG and nanosilicate (nSi)	No cells	Intrabony defects in the mandible	Wistar rats 1, 2, 4, 8 weeks	Comparative angulation of fiber orientation of the developed PDL to native PDL and thicker cementum formation
Daghrery 2021 et al. [12]	PCL scaffolds were fabricated via Melt ElectroWriting (MEW) and were subsequently applied to F/CaP coating process	No cells	Periodontal defect model with fenestration defects bilaterally in the mandible.	Fischer 344 rats 3 and 6 weeks	Bone formation after 3 and 6 weeks significantly enhanced in F/CaP-coated scaffolds. Regeneration of new alveolar bone, cementum, and PDL even after 3 weeks and connective tissue fibers orientation similar to normal PDL
Yu 2022 et al. [11]	Bilayer construct: self-assembly and microstamping strategies IMC scaffold with CGF	No cells	Periodontal defect model with fenestration defects in mandibular 1st molars	Sprague-Dawley rats 8 weeks	Regeneration of both mineralized (cementum and bone) and non-mineralized soft connective tissues (PDL) with structure and fibrous orientation similar to normal PD.

**Table 3:** Clinical studies involving scaffolds and growth factors for periodontal tissue regeneration.

Author, Year	Type of Scaffold	Research Type	Experimental Groups	Number of Subjects, Term	Outcome Measurements	Results
McGuire & Scheyer 2006 [161]	β-TCP in combination with rh-PDGF-BB and collagen membrane	Clinical case series	Patients with recession defects > 3 mm, in contralateral quadrants of maxilla, excluding molars: (1) rhPDGF-BB and collagen membrane and β-TCP (2) subepithelial connective tissue graft (CTG)	7 patients, up to 24 weeks	Clinical measurements of recession depth	The use of new graft material has comparable results to CTG method in treatment of gum recession.
Sarment et al., 2006 [162]	β-TCP in combination with rh-PDGF-BB	Clinical study	Patients with vertical bone defects: (1) β-TCP (active control *n* = 15), (2) β-TCP + 0.3 mg/mL of rhPDGF-BB (*n* = 14), or (3) β-TCP + 1.0 mg/mL of rhPDGF-BB (*n* = 18).	47 patients, 24 weeks	Wound fluid analysis by radioimmunoassay for pyridinoline crosslinked carboxyterminal telopeptide of type I collagen (ICTP)	Increase in the amount of ICTP up to 6 weeks was detected in the 0.3 and 1.0 mg/mL PDGF-BB treatment groups, indicating bone turnover
McGuire et al., 2006 [163]	rhPDGF-BB with synthetic β-TCP	Clinical case series	Group 1: sites treated with 0.3 mg/mL rhPDGF-BB + β-TCP. Group 2: beta-TCP with buffer solution (control)	4 patients, 24 months	Clinical and radiographic parameters	Significant improvements of clinical and radiographic parameters in sites treated with rhPDGF-BB + β-TCP.
Bhongade & Tivari, 2007 [164]	Type-I collagen and cell binding peptide (P-15) with anorganic bovine matrixABM	Clinical study	Test group: OFD with a bovine-derived xenograft enriched with a cell binding peptide P-15, Control group: only OFD	20 interproximal intraosseous defects in 16 patients, 6 months	Clinical and radiographic assessments	Experimental group demonstrated significantly increased mean defect fill.
McGuire et al., 2009 [165]	β-TCP in combination with rh-PDGF-BB and collagen dressing	Randomized control trial	Patients with Miller Class II buccal gingival recession, >3 mm: (1) rhPDGF-BB and collagen dressing and β-TCP, (2) subepithelial connective tissue graft (CTG)	30 patients, 6 months	Histologic/micro-CT	Evidence of regeneration of bone, cementum and PDL with connective tissue fibers insertion, whereas neither CTG-treated site exhibited any signs of periodontal regeneration
Jayakumar et al., 2011 [166]	rhPDGFBB and β-TCP	Multi-centre, randomized clinical trial	Two groups with moderate and advanced periodontitis: (1) β-TCP graft with rhPDGF-BB (*n* = 27) (2) β-TCP (control, *n* = 27).	54 patients, 3 and 6 months	Clinical and radiographic parameters (linear bone growth (LBG) and percent bone fill (%BF)	Significantly higher linear bone growth and percent bone fill in the experimental group
Nevins et al., 2013 [167]	β-TCP scaffold matrix and PDGF-BB	Multicenter, randomized, controlled clinical trial	Three groups: (1) (β-TCP) (scaffold) with sodium acetate buffer alone; (2) β-TCP with 0.3 mg/mL rhPDGF-BB; and (3) β-TCP with 1.0 mg/mL rhPDGF-BB in patients with advanced periodontal defects.	135 patients, 36 months	Clinical and radiographic evaluation	rhPDGF-BB at 0.3 mg/mL resulted in significantly greater clinical and radiographic finfings, in moderate to severe 2- and 3-wall periodontal intrabony defects
Maroo & Murthy, 2014 [168]	β-TCP in combination with rh-PDGF-BB	Randomized clinical trial	30 sites were randomly divided into test group (β-TCP) in with rh-PDGF-BB and control group-only β-TCP	15 patients, 9 months	Clinical and radiographic examination	Sites with rhPDGF + β-TCP demonstrated significantly greater reduction of pocket depth and gain in clinical attachment level. Significantly higher amount and percentage of defect fill in test sites.
Rasperini et al., 2015 [169]	3D printed PCL and 4% of HA scaffold. Internal part: pegs for PDL guidance and compartment for rhPDGF-BB delivery	Clinical case report	Scaffold implantation in region of #43 tooth	1 patient, 14 months	Clinical examination	The scaffold remained covered for 12 months but was removed after exposure after 13 months. Clinical partial root coverage and 3 mm attachment gain were observed. No signs of chronic inflammation or dehiscence.
Hamzacebi et al., 2015 [170]	PRFmembrane and plug	Clinical study	Two groups: (1) patients who recieved PRF scaffold and (2) (control) patients who received only the access flap.	19 patients with peri-implant bone loss, 6 months	Clinical assessment	Significantly higher mean reduction of probing depth and clinical attachment gain compared to the control.
Kitamura et al., 2016 [171]	Hydroxypropyl cellulose with rhFGF-2	Multicenter, randomized clinical trial	Study A. Patients with advanced periodontitis received 0.3% rhFGF-2 or Placebo after flap surgery Study B. Patients received rhFGF-2, enamel matrix derivative (EMD) therapy, or flap surgery.	Study A: 328 patients Study B: 274 patients, 36 weeks	Serum antibodies measurement, clinical and radiographic data	Study A: significantly higher percentage of bone fill in the rhFGF-group, no significant differences in clinical attachment level between groups. Study B: significantly higher linear alveolar bone growth in rhFGF-2 group as compared to EMD group in the EMD group, efficacy of rhFGF-2 treatment in smokers.
Cochran et al., 2016 [172]	β-TCP loaded with rhFGF-2	Double-blinded, dose-verification, externally monitored clinical study	Patients with vertical bone defects: 1 Group: β-TCP alone (control) 2 Group: β-TCP + 0.1% rh-FGF-2, 3 Group: β-TCP + 0.3% rh-FGF-20.3% 4 Group: β-TCP + 0.4% rh-FGF-2	88 patients, 6 months	Clinical and radiographic evaluation	Groups 3 and 4 showed significant clinical improvements as compared to others.
Naineni et al., 2016 [173]	β-TCP loaded with Alendronate (ALN),	Randomized prospective clinical study	Patients with vertical periodontal defects (>4 mm): (1) 400 μg ALN + β-TCP + Saline (test) and (2) β-TCP + Saline (active-control).	32 patients, 6 months	Clinical and radiographic evaluation	Experimental scaffold improved soft tissue parameters, inhibited alveolar crestal resorption and enhanced bone formation, compared to β-TCP
Khan et al., 2017 [174]	Tinidazole (TNZ) functionalized biodegradable chitosan/PCL mucoadhesive hybrid nanofiber membrane	Preliminary clinical trial	3 periodontal sites in patients with chronical periodontitsis: (1) scaling and root planning; (2) placebo and fiber; (3) medicated nanofiber	10 patients, 8 weeks	Clinical examination	Significant decrease in clinical markers of periodontitis in the experimental group
Lee et al., 2017 [175]	Equine-derived bone matrix vs. β-TCP with rh-PDGF-BB	A single blinded comparative study	Patients with advanced periodontitis: (1) rhPDGF-BB + equine-derived bone matrix, (2) rhPDGF-BB + β-TCP (control)	32 patients, 6 months	Clinical examination and X ray	Group 1 showed significant CAL gain. No statistically significant change in radiographic bone level betweengroups.
Deshoju et al., 2017 [176]	Zn-substituted monetite-based scaffold	Randomized controlled clinical trial (split-mouth, double-blind)	Patients with chronical periodontitis and vertical bone loss: (1) open flap debridement (OFD) + Sil-Oss^®^, (2) OFD + HA bone graft (control).	30 patients, 9 months	Clinical and radiographic analysis. Histological evaluation after 7 months (bone biopsy)	No significant differences in clinical parameters between groups after 6 months and significant increase in bone fill of the experimental material as compared to HA.
Kizildag et al., 2018 [177]	Leukocyte-PRF membrane	Randomized controlled trial	Patients with chronic periodontitis with horizontal bone loss, were treated by OFD alone (control) or L-PRF + OFD.	16 patients, 6 months	Clinical examination and biochemical detection of levels of growth factors in gingival crevicular fluid	Significantly higher PD reduction and CAL gain were observed in the L-PRF treated sites and increased BMP-2 and IGF-1 at 2 weeks.
Saito et al., 2019 [178]	(rhFGF)-2 REGROTH^®^ with deproteinized bovine bone mineral (DBBM)	Randomized clinical trial	Patients with moderate to severe chronic periodontitis: (1) 0.3% rhFGF-2 + DBBM (*n* = 22) and (2) rhFGF-2 alone (*n* = 22, control).	Number of patients not stated 6 months	Clinical and radiographic parameters	No significant difference in clinical attachment gain was observed, improved radiographic outcome in Group 1.
Lee et al., 2020 [179]	EMD in combination with demineralized porcine bone matrix (DPBM)	Randomized controlled clinical trial.	Patients with one-wall intrabony defects in the molar regions: (1) DPBM + EMD (*n* = 20), (2) DPBM (control, *n* = 22).	42 patients, 24 months	Clinical and radiographic parameters	No severe adverse effects, no statistically significant differences between groups, better wound healing in Group 1.
Shoukheba et al., 2021 [180]	β-TCP gelatin sponge soaked in CGF	Randomized clinical trial	Patients with moderate and severe periodontitis: (1) surgery plus biodegradable gelatin/β-TCP sponges, (control group, *n* = 10), (2) gelatin/β-TCP sponges socked in CGF(*n* = 10).	20 patients, 6 months	Clinical examination, CBCT (bone defect area and density)	Investigated scaffolds enhanced the outcome of periodontal regeneration, as evidenced by improved bone density and reduction in the defect area.
Mangano et al., 2021 [181]	30% HA-70% β-TCP BCP 3D-printed scaffold,	Case report	Implantation of 3D-printed biphasic-HA block and implant placement in the area of #15 (unloaded) in 2013 and bridge placement in the area 14–17	1 patient, 7 years	Micro CT and histomorphometrical analyses.	Regeneration of lamellar bone, scaffold integration and signs of scaffold degragation. 57% of newly formed bone detected by micro-CT.
Deshpante et al., 2021 [182]	ECM component—natural collagen to nHA bone graft	Randomized controlled clinical study	Group 1: nHA + natural collagen (20 sites) Group 2: nHA with natural collagen and Group B: nHA (20 sites)	40 patients, 3 and 6 months	Clinical and radiographic evaluation.	Statistically significant improvement in clinical attachment indexes was noted in Group A after 3 months, while the results were similar in both groups after 6 months.
Venkatesan et al., 2021 [183]	Amniotic membrane or porcine collagen membrane with Biphasic calcium phosphate BCP(60% HA 40% β TCP).	Randomized clinical study	Patients with localized moderate to severe periodontitis: (1) Collagen membrane + BCP and (2) Amniotic membrane + BCP.	50 patients, 6 months	Radiographic bone fill and clinical measurements	No statistically significant difference between the groups

**Table 4:** Clinical studies involving cells and scaffolds for periodontal tissue regeneration.

Author, Year	Type of Scaffold	Type of Cells	Research Type	Experimental Groups	Number of Subjects, Term	Outcome Measurements	Results
Mohammadi et al., 2007 [195]	Collagen gel	hGF	Climical study	hGF from attached gingiva was added to collagen gel. Each patient: 1 tooth treated with a periosteal fenestration technique (control group) or a tissue engineered mucosal graft (test group).	9 patients (18 sites), 3 months	Clinical parameters: width of keratinized tissue, probing depth, and width of attached gingiva	The mean amount of attached gingiva was significantly higher at test sites than at control sites.
Jhaveri et al., 2010 [198]	Acellular dermal matrix allograft (ADMA) seeded with autologous GFs	autologous hGF	Split-mouth, controlled, double-masked clinical case series	Patients with Class I or II recessions of maxillary canines or premolars: subepithelial connective tissue graft (control group) or an ADMA seeded with autologous GFs (test group).	10 patients, 3 and 6 months	Clinical parameters, healing time and inflammation were assessed.	No significant differences between test and control sites. The test sites showed less inflammation in the early postoperative period
Dhote et al., 2016 [188]	(β-TCP) in combination with rh-PDGF-BB	Umbical cord MSCs	Randomized Clinical trial	The control group (*n* = 12 sites) was treated by an open flap debridement (OFD) only, while the test group (*n* = 12 sites) was treated by a MSCs cultured on β-TCP in combination with rh-PDGF-BB.	14 patients with moderate to advanced periodontitis, 6 months	Clinical measurements and radiographic analysis	Significant improvrmrnts in clinical parameters & greater radiographic defect depth reduction and defect fill in test group.
Chen et al., 2016 [191]	Bio-Oss	Autologous PDLSCs	Single center, randomized clinical trial	Group 1: GTR and PDLSC + Bio-oss^®^ and Group 2: GTR + Bio-oss^®^ (control group).	30 patients, 12 months	Clinical and radiographic evaluation	No statistically significant differences were detected between groups.
Baba et al., 2016 [190]	biodegradable three-dimensional (3D) woven fabric PLLA resin scaffold and PRP	Iliac bone marrow autologous MSC	phase I/II clinical study	Implantation of scaffolds with MSC and PRP, no control group	10 patients, 36 months	Clinical parameters, laboratory tests of blood and urine samples	Improvement of all clinical parameters during the entire follow-up period.
Aramoon et al., 2017 [197]	PRGF	Autologous hGF	Pilot clinical study	Patients with gingival recession: (1) Periosteal fenestration on one side (control) and (2) tissue-engineered mucosal graft (test)	4 patients (8 sites), 3 months	Probing depth (PD), width of keratinized and attached gingiva	Significantly incerased width of keratinized gingiva in test group.
Iwata et al., 2018 [8]	β-TCP	Autologous PDL-erived cell sheets	A single-arm and single-institute clinical study;	3-layered PDL-derived cell sheets were fabricated and applied to bone defects with β-TCP granules.	10 patients, 6 months and 55 ± 19 months follow up	Clinical examination and CBCT	Improvement of clinical parameters and increase of bone height.
Hernandez-Monjaraz et al., 2018 [199]	lyophilized collagen-polyvinylpyrrolidone sponge	MSCs from dental pulp of a deciduous tooth	Case report	Tooth #35 with pocket depth 6.5 mm and II stage of mobility, underwent flap surgery and scaffold placement	1 patient, 6 months	Clinical and radiographic evaluation	Decrease in tooth mobility, periodontal pocket depth and bone defect area.
Ferrarotti et al., 2018 [189]	Collagen sponge	Autologous DPSCs	Randomized controlled trial	Patients with severe periodontitis: (1) DPSC micrografts seeded onto collagen sponge (*n* = 15), (2) collagen sponge alone (*n* = 14, control).	29 patients, 12 months	Clinical and radiographic evaluation	Significantly greater clinical attachment level gain and bone defect fill in test groups.
Abdal-Wahab et al., 2020 [62]	β TCP	Autogenous hGF and associated mesenchymal stem cells (GMSC)	Randomized controlled clinical trial and biochemical study	Patients with advanced periodontitis: (1) β-TCP +collagen membrane (*n* = 10), (2) β-TCP scaffold with seeded GF and collagen membrane (*n* = 10)	20 patients, 6 months	Clinical and CBCT examination, quantitative measurement of PDGF-BB and BMP-2 in gingival crevicular fluid.	Significant improvements in clinical measurements in the test group. Statistically higher radiographic bone gain in the test group and higher concentration of PDGF-BB on days 1, 3, and 7.
Kashte et al., 2020 [200]	PCL-GO-Cissus quadrangularis (CQ)(PCL-GO-CQ) scaffold	Human umbilical cord Wharton’s jelly derived MSCs	Case report	Multiple gingival recessions (Miller’s class II)	1 patient, 2 months	Clinical examination	Significant reduction of gingival recession with over 70% of root coverage
Sanchez et al., 2020 [192]	Xenogeneic bone substitute (XBS)	PDL-MSCs	quasi-randomized controlled pilot clinical trial	Patients with moderate and severe chronic periodontitis with one or two wall defects: (1) XBS + PDLMSCs and (2) XBS (control)	20 patients, 12 months	Clinical and radiographic evaluation	No significant differences between groups, low morbidity, and safety of cell-based therapyll-based therapy
Apatzidou et al., 2021 [193]	Collagen scaffolds with autologous fibrin/platelet lysate (aFPL).	Autologous alveolar bone marrow MSCs	A proof-of-principle randomized clinical study	Group-1 (*n* = 9) BMMSCs seeded into collagen scaffolds, and aFPL. Group-2 (*n* = 10), the collagen scaffold/aFPL seeded with a BMMSCs Group-3 (*n* = 8) no scaffold, minimal access flap surgery	27 subjects with advanced periodontitis, 12 months	Radiographic bone fill and clinical measurements	Significant clinical improvements with no inter-group differences Better clinical outcomes in Groups 1 and 3, over 2nd.

## Data Availability

All data are reported in tables.

## Supplementary Materials

The following supporting information can be downloaded at: https://www.mdpi.com/article/10.3390/biom12030435/s1, Table S1: In vivo studies employed orthotopic models to assess the regenerative capacity of cell sheet transplantation in periodontal defect models, with or without biomaterials; Table S2: In vivo studies evaluating scaffolds for PDL regeneration employing the subcutaneous implantation model; Table S3: In vivo studies evaluating scaffolds for PDL regeneration employing other models than periodontal defect and subcutaneous placement model.

## Abbreviations

ABM	Inorganic bovine matrix
ACP	Amorphous calcium phosphate
Ad-BMP-7, AdBMP7	Recombinant adenovirus-encoding murine bone morphogenetic protein-7
AdPDGF-B	Adenovirus for platelet-derived growth factor-B
AEFC	Acellular extrinsic fiber cementum
ALP	Alkaline phosphatase
APCs	alveolar periosteal cells
BFGF	Basic fibroblast growth factor
BGC	Bioactive glass ceramic
BMP-2	Bone morphogenetic protein 2
BMSCs, Bm-MSCs, BMMSCs	Bone marrow mesenchymal stem cells
CBB	ceramic bovine bone
CCRD	chemical conditioned root dentin
CEMP1	Cementum matrix protein 1
CGF	Concentrated growth factor
CHI	Chitosan
CIFC	Cellular intrinsic fiber cementum
CMSC	Cellular mixed stratified cementum
Col	Collagen
CTGF	Connective tissue growth factor
DBBM	Deproteinized bovine bone mineral
DDM	decalcified dentin matrix
DePDLSCs	PDLSCs from deciduous teeth
DFSCs, DFCs	Dental follicle stem cells
dHPDLC sheet	Decellularized human periodontal ligament cell sheet
DMOG	Dimethyloxalylglycine
dMSCs	Dog mesenchymal stem cells
dPDLCs	Dog periodontal ligament cells
DPEM	Dental pulp extracellular matrix
DPSCs	Dental pulp stem cells
DSCs	Epithelial dental stem cells
DTT	Dithiothreitol
ECM	Extracellular matrix component
EMD	Enamel matrix protein derivative
ePTFE	Polytetrafluoroethylene
FACP	Fluorine containing amorphous calcium phosphate
FDM	Fused Deposition Modeling
FGF-2, FGF2	Fibroblast growth factor 2
FN	Fibronectin
GC	Gingival cells
Gel-MA	Gelatin methacrylate
GEN	Genipin
G-MSCs	Gingival margin-derived stem/progenitor cells
GO	Graphene oxide
HA	Hydroxyapatite
HA/TCP	Hydroxyapatite/tricalcium phosphate
hGFs	Human gingival fibroblasts
HGF	Hepatocyte growth factor
hJBMMSCs/hBMMSCs	Human (Jaw) bone marrow-derived mesenchymal stem cells
hOB	Human osteoblasts
HPDLs/HPDLCs	Human periodontal ligament primary cells
HPDLSCs	Human periodontal ligament stem cells
HPDLSCs	Periodontal ligament stem cells from healthy donors
HUVECs	Human umbilical vein endothelial cells
IGF2	Insulin growth factor 2
IMC	Intrafibrillarly mineralized collagen
IPCs	Induced pluripotent stem cells
JBMMSCs	Jawbone marrow mesenchymal stem cells
L-PRF	Leukocyte-PRF
LIPUS	low-intensity pulsed ultrasound
MBCP	micro/macro-porous biphasic calcium phosphate
MCPs	Monolayered cell pellets
MCS	Monolayered cell sheet
MBCP	Micro/macro-porous biphasic calcium phosphate
MUCPs	Multilayered cell pellets
MUCS	Multilayered cell sheet
OFD	Open flap debridement
PCL	Polycaprolactone
PCL/GE	Polycaprolactone/gelatin
PDGF	Platelet-derived growth factor-B
PDL	Periodontal ligament
PDLCs	Periodontal ligament cells
PDLSCs, PDL-MSCs	Human periodontal ligament stem cells
PEG	Poly(ethylene glycol)
PEG-DA	Polyethylene glycol diacrylate
pFGF-2	Plasmid DNA encoding fibroblast growth factor-2
PePDLSCs	Periodontal ligament stem cells from permanent teeth
PGA	Polyglycolic acid
PisPLLA	Polyester poly(isosorbide succin-ate-co-L-lactide)
PLAP-1	Periodontal ligament-associated protein-1
PLGA	Poly-DL-lactic-co-glycolic acid
PLLA	Poly(L-lactide)
PPD	Pocket probing depth
PPDLSCs	Periodontal ligament stem cells from patients with periodontitis
PRF	Platelet-rich fibrin
PRGF	Platelet-rich in growth factor
PRP	Platelet-rich plasma
rh-AM, rhAM	Recombinant human amelogenin
rh-BMP-2	Human recombinant bone morphogenetic protein 2
rhCEMP1	Recombinant human Cementum matrix protein 1
rhFGF-2	Recombinant human Fibroblast growth factor 2
rh-PDGF-BB	Recombinant human Platelet-Derived Growth Factor-BB
SCAPs	Stem cells from apical papilla
SDF-1	Stromal cell-derived factor-1
SDF-1	Stromal cell-derived factor-1
SHEDs	Stem cells from human exfoliated deciduous teeth
TDM	Treated dentin matrix
Ti	Titanium
USCs	Urine-derived stem cells
Vc	Vitamin C
XBS	Xenogeneic bone substitute
β-TCP	β-tricalcium phosphate
