# Supplementary material for: A Review of In Vivo and Clinical Studies Applying Scaffolds and Cell Sheet Technology for Periodontal Ligament Regeneration
Source: Biomolecules. 2022 Mar 11;12(3):435. doi: 10.3390/biom12030435 (PMC8945901; doi:10.3390/biom12030435)
Supplement: Supplementary file 1 [file biomolecules-12-00435-s001.zip › biomolecules-1607320-supplementary.pdf]

Supplementary Table S1. In vivo studies employed orthotopic models to assess the regenerative capacity of cell sheet transplantation in periodontal defect models, with or without biomaterials

Author	Cells	Experimental groups	Additional pretreatment	Material	Technique	Experimental setup	Results
Yang et al., 2009[1]	hPDLSCs, APTGs	APTGs-CM treated PDLSC pellets with CBB/CCRD groups, untreated PDLSC pellets/CBB/CCRD group, CBB/CCRD without cells group	Culture with APTGs-CM	CBB and CCRD	Culture in CM, and cell sheet to cell pellet	Ectopic (subcutaneous) implantation in mice	Histological analysis of the APTG-CM treated group showed cementum-like and PDL-like tissues, whereas histological analysis of the untreated control group sporadically revealed cementum/PDL-like tissues.
Washio et al., 2010[2]	hPDLCs	PDL cell sheets	None	PGA film and dentin block	Cell sheet	Triple-layered cell sheets combined with human dentin blocks implanted subcutaneously onto the back of nude mice	Histological analysis revealed newly formed PDL-like tissue and cementum around dentin blocks in all samples.
Xie & Liu, 2012[3]	hPDLSCs,h BMMSCs	Co-cultured pellet group, non-co-cultured pellet group	Co-culture	CBB	Co-culture and mixed with CBB, cell sheet	Mixed-type sheet turned into stem cell pellet and transplanted into nude mice	Co-cultured pellet group promoted the regeneration of cementum-like and PDL-like tissue, along with angiogenesis, whereas non-co-cultured pellet group exhibited cementum-like tissue regeneration to a limited extent.

Ji et al., 2012 [4]	DePDLSCs, PePDLSCs	DePDLSCs group, PePDLSCs group	None	Dentin block	Cell sheet	Cell sheets on dentin blocks transplanted into the peritoneal cavities of nude mice	Both cell sheets resulted in PDL-like tissue formation, when transplanted in vivo. However, cementum-like tissue was developed only in the DePDLSCs group.
Wei et al., 2012[5]	PDLSCs	Vc-induced autologous PDLSCs sheet group, UpCell dish PDLSCs sheet group, Gelfoam scaffolds/dissociated autologous PDLSCs group (control)	Vc treatment	Gelfoam scaffold	Cell sheet	Ectopic transplantation in nude mice, and orthotopic transplantation in experimental periodontal lesions in miniature swines	Vc-induced PDLSCs sheet group and UpCell dish PDLSC sheet group application resulted in significantly more bone/cementum-like tissue formation compared to the control group, and Vc-induced PDLSCs sheet group performed significantly better compared to UpCell dish PDLSCs sheet group.
Yang et al., 2012[6]	DFCs	DFCSs/TDM group, DFCSs/HA/TCP group, TDM containing no cells group, DFCSs group		HA/TCP and TDM	Cell sheet	Subcutaneous implantation into the dorsum of nude mice	TDM was more beneficial on DFCs towards promoting cementum-like and PDL-like tissue, thus supporting root-PDL complex regeneration.

Guo et al., 2013[7]	PDLSCs,DFCs	PDLSC sheets groups, DFC sheets group	None	TDM	Cell sheet	Sheet/TDM complexes were implanted subcutaneously into nude mice	DFC and PDLC sheets both exhibited regenerative potential towards PDL-like tissue, however DFCs sheets showed superior behavior in terms of PDL-like tissue regeneration.
Wei et al., 2013[8]	autologous and allogeneic PDLSCs	HA/TCP group; autologous Vc-induced PDLSCs sheet wrapping the HA/TCP/DPSC group, allogeneic Vc-induced PDLSCs sheet wrapping the HA/TCP/DPSC group	Vc treatment	HA/TCP	Cell sheet	Implantation into jawbone implant sockets in swine	Radiographic and histological analysis revealed successful PDL-like tissue formation in both groups, after six months of in vivo implantation.

Liu et al., 2014[9]	HPDLSCs, PPDLSCs, DFCs	Co-culture DFCs/HPDLSCs group, co-culture DFCs/PPDLSCs group	Co-culture between PDLSCs and DFCs	CBB and CCRD	Cell sheet of co-cultured cells	Subcutaneous pockets in mice	Histological analysis of the DFCs/HPDLSCs group revealed a PDL-like tissue regeneration closely integrated into the CBB and CCRD surfaces. Whereas, in the DFCs/PPDLSCs group, there were many inflammatory cells present in the regenerated tissue, and newly formed PDL-like tissue did not adhere well to the CBB and CCRD surfaces.
Xu et al., 2014[10]	PDLSCs	Control group, 1% PRP group	PRP	HA/TCP ceramic powders	Cell sheets combined with HA/TCP	Ectopic transplantation with HA/TCP in nude mice	Histological analysis of in vivo transplantation showed the 1%PRP group demonstrated superior periodontal tissue regenerative potential when compared with the control group.
Guo et al., 2014[11]	Rat PDLSCs	MCPs group, MUCPs group, MCPs/TDM group, MUCPs/TDM group	None	TDM	MCP and MUCPs formed by MCS and MUCS	In vivo transplantation into the omental pouch; and in a periodontal defect model in rats	Following in vivo transplantation, all groups promoted cementum-like and PDL-like tissue regeneration, but MUCPs group exhibited superior behavior in terms of mineralization and collagen

							fiber arrangement compared to MCPs group.
Gao et al., 2015[12]	PDLSCs, BMMSCs	Different nanotubes, NT5, NT10, and NT20		Titania nanotubes (NTs) layered on Ti	Cell sheet	ectopic implantation model using a Ti/cell sheets/HA complex in nude mice	DPSCs sheets in combination with BMMSCs sheets were able to regenerate PDL-like tissues on the Ti surface, when implanted in vivo.
Zhang et al., 2016[13]	PDLSCs, JBMMSCs	Cell sheets composed of hPDLSCs, hJBMMSCs and the mixed cells of the two	Co-culture between PDLSCs and JBMMSCs	hTDM and CBB	Cell sheet and co-culture and use of hTDM and CBB	Ectopic transplantation with CBB and hTDM in nude mice	Mixed stem cell sheet exhibited superior behavior in terms of PDL-like tissue regeneration compared to the PDLSC and JBMMSC sheets.
Panduwawala et al., 2016[14]	PDLSCs, HUVECs	Control group, triple cell sheet of PDLSCs group, PDLSCs-HUVECs-PDLSCs group, cell sheets of co-cultures group	Co-culture		Triple cell sheet of co-cultured cells	Cell sheets combined with human roots implanted subcutaneously into nude mice.	PDL-like tissue regeneration and angiogenesis was observed in the PDLSCs-HUVECs-PDLSCs group and the cell sheets of co-culture group.

Yu et al., 2016[15]	PDLSCs	Inflammation group, hypoxia group, inflammatory plus hypoxic stimuli-dual-stimuli group, no-stimulus group, blank group, CBB group	Inflammatory conditions, hypoxic conditions, or a combination of both conditions	CBB	Cell sheet	Ectopic transplantation model (subcutaneously) into the dorsal region, and orthotopic model with surgical creation of periodontal defects (3 mm × 1.5 mm) in nude mice	Radiographic analysis showed that hypoxia group presented increased bone formation compared to the other groups. Control group presented less bone formation compared to inflammation group and dual-stimuli group. Histological analysis showed that hypoxia group exhibited more bone formation, while cementum-like and PDL-like tissue formation was identified in the control and hypoxia groups.
Wang et al., 2016[16]	PDLSCs, JBMMSCs	PDLSC sheets/PRF/PDLSC sheets group, JBMSC sheets/PRF/JBMSC sheets group, PDLSC sheets/PRF/JBMSC sheets group	PRF onto bioabsorbable fibrin scaffolds containing growth factors	PRF fabricated into bioabsorbabl e fibrin scaffold, and TDM and HA/TCP frameworks	Cell sheet	Subcutaneous transplantation of PDLSC sheet/PRF/JBMSC sheet composites combined with TDM and HA/TCM in nude mice	PDLSC sheets/PRF/PDLSC sheets group promoted PDL-like tissue formation, whereas JBMSC sheets/PRF/JBMSC sheets group promoted bone tissue regeneration. PDLSC sheets/PRF/JBMSC sheets group supported the regeneration of PDL-like and bone tissue, thus presenting the most promising periodontal regenerative potential.

Gao et al., 2016[17]	PDLSCs	Engineered bio-root and dental implants	Vc treatment	HA/TCP scaffolds	Cell sheets combined with HA/TCP	HA/TCP/DPSC/PDLSC sheet complex which was implanted into the implant socket in miniature pig	Engineered bio-roots promoted PDL-like tissue formation, as depicted by the histological analysis. Engineered bio-roots exhibited biochemical properties similar to those of natural tooth roots, but lower success rate when compared to dental implants.
Feng et al., 2017[18]	hDFCs	dentin + hDFCs - DDM sheet + HA-TCP group, dentin + hDFCs – PLGA sheet + HA-TCP group, dentin + hDFCs sheet+ HA-TCP group	None	frozen porous DDM sheets and HA/TCP	Cell sheets combined with DDM and HA/TCP to produce a sandwich like structure	Ectopic model in nude mice	Histological analysis showed superior results for the experimental group (dentin + hDFCs - porous DDM sheet + HA-TCP group), which promoted PDL-like tissue formation through collagen production and deposition, while immunohistochemical analysis revealed the presence of OPN and periostin.
Zhang et al., 2017[19]	PDLSCs	PDLSC-sheets from plastic surfaces group, PDLSC-sheets expanded on the produced ECM group	None	TDM	Multilayered cell sheets	Ectopic model (subcutaneous transplantation) in nude mice	Both groups exhibited regenerative potential towards PDL-like tissue. Histological analysis showed superior behavior of the PDLSC-sheets expanded on ECM in terms of thickness and structure of the

							regenerated PDL-like tissues.
Washio et al., 2018[20]	hPDLcells	Commercially pure Ti group, Ti treated with acid etching group, Ti treated with blasting, Ti treated with a calcium phosphate coating group	None	Ti	Periodontal ligament cell sheet on different implant surfaces	Transplantation of Ti/cell sheets in bone marrow cavity in athymic rats, transplantation of Ti/with or wo cell sheets, in mandibular bone defect in beagle dogs	Ti surface treatments promoted cementum-like and PDL-like tissue regeneration onto the Ti surface, with perpendicular collagen fiber orientation.
Yang, Ma et al., 2019[1]	DFCs, SHEDs	SHEDSs/TDM group, DFCSs/TDM group, TDM	None	TDM	Cell sheet	Subcutaneous transplantation into nude mice and orthotopic implantation in Sprague-Dawley rats' jawbone	Histological analysis revealed that both SHEDs/TDM and DFCs/TDM groups formed PDL-like tissues, enriched in collagen fibers and fibroblasts, with arrangement similar to that of native PDL, exhibiting promising periodontal regenerative potential.
Hu et al., 2019[21]	DPSCs, SCAPs, PDLSCs	DPSCs sheet group, SCAPs sheet group, PDLSCs sheet group	Vc treatment	HA/TCP	Cell sheet combined with HA/TCP	Ectopic model (subcutaneous transplantation) in nude mice	DPSC sheet group promoted the regeneration of pulp-like tissue, rich in connective tissue fibers and vessels. PDLSC sheet group promoted the regeneration of PDL-like tissue, rich in directionally oriented

							collagen fibers, whereas SCAP sheet group promoted the regeneration of mineralized tissue.
Yang et al., 2020[22]	hPDLSCs, USCs	group 1: 1:0.5 (1 × 105 PDLSCs and 0.5 × 105 USCs), group 2: 1:1 (PDLSCs and USCs), group 3: 1:2 (PDLSCs and USCs). As a control, 1 × 105 PDLSCs were also cultured in a monolayer	Co-culture at different ratios	HA	Cell sheet	Ectopic transplantation in nude mice of wrapped HA surrounded by three-layered cell sheets	Histological and immunohistochemical analysis revealed that group 3 (PDLSC/USC 1:2 ratio) promoted the regeneration of bone and cementum-like tissues compared to the other groups.
Li et al., 2020[23]	hPDLSCs	HA group, HA + cell suspension group, hPDLSC sheets group + HA, LIPUS-treated hPDLSC sheets group + HA	LIPUS	HA	Cell sheet combined with HA	Ectopic model (subcutaneous transplantation) in nude mice	Histological analysis revealed PDL-like tissue regeneration in the hPDLSCSs group and the LIPUS-treated group. LIPUS-treated group exhibited increased collagen deposition and COL-1, OCN, and periostin expression compared to the other groups.

Zhao et al., 2020[24]	PDLSCs	PCL and PCL-SIM	None	PCL membrane scaffold ±SIM, root dentin and CBB	Cell sheet	Multilayers PDLSC sheets on PCL or PCL–SIM scaffolds were placed between dentin and CBB for subcutaneous implantation in athymic mice	Histological analysis revealed that PCL-SIM scaffold enhanced the regenerative potential of PDLSC sheets, promoted the formation of mineralized tissue and collagen fiber deposition, leading to the regeneration of cementum-like and PDL-like tissue compared to the PCL scaffold.
Park et al., 2020[25]	hPDLSCs	MBCP block group, hPDLSC sheet-MBCP block group, rhBMP-2-hPDLSC sheet-MBCP block group	rhBMP-2 pre-treatment	MBCP blocks	Cell sheet combined with MBCP block	Ectopic model (subcutaneous transplantation) in nude mice	rhBMP-2-hPDLSC sheet application in vivo revealed mineralized tissue production and collagen deposition, showing great regeneration potential towards PDL-like and cementum like tissue formation compared to the hPDLSC sheet group that did not receive rhBMP-pretreatment.
Meng et al., 2020[26]	hDPScs	subcutaneous transplanted sandwich structure (Regenerated), TDM fabricated from native tooth	Vc treatment	hTDM and matrigel	Cell sheet combined with TDM and Matrigel	Ectopic model of subcutaneous transplantation of sandwich structure of hDPSC sheet/TDM/Matrigel in nude mice	The sandwich implant of hDPSC sheet/hTDM/Matrigel developed into a tooth root like structure in vivo. Outside of the sandwich implant, periodontium-like paralleled fibers were observed, which

		root (TDM), native human tooth root (Native)					contained fibroblast like cells.
Raju et al., 2020[27]	rat PDL cells and osteoblastic cells	PDL cell sheet group, MC3T3-E1 cell sheet group, complex cell sheet containing both cells group	None	None	Cell sheet	Ectopic and orthotopic transplantation in mice	Ectopic transplantation of complex cell sheet resulted in PDL-like and bone tissue formation, which was validated through the identification of periostin and osteocalcin expression. Histological analysis of orthotopic transplantation indicated that only complex cell sheet group was able to regenerate bone and PDL-like tissue similar to the native PDL-bone complex.

Supplementary Table S2. In vivo studies evaluating scaffolds for PDL regeneration employing the subcutaneous implantation model.

Study	Scaffold type	Cells	Animal/ Evaluation time	Major findings
Park 2010 et al.,[28]	Multi-scale composite hybrid PCL-PGA scaffolds for PDL and bone portions	BMP-7- modified hGFs in the bone region and hPDL cells in the PDL interface	Immunodeficient NIH III nude mice 6 weeks	The cementum-like tissue formed at greater extends of the hPDL/BMP-7- seeded scaffolds while almost no cementum-like tissue was observed in the other groups. Vascularized fibrous connective tissues in close proximity with cementum-like tissues
Vaquette 2012 et al.,[29]	Biphasic electrospun scaffold PCL + β-TCP 20% wt] sutured on dentin slices	Scaffolds seeded or not with osteoblasts and PDL cell sheets	Athymic nude rat 8 weeks	Ectopic periodontal regeneration, fibers' attachment was observed in the cell sheets groups with cementum-like tissue on the dentin surface
Yang 2012 et al.,[6]	Biphasic HA/TCP scaffold and human TDM	DFCSs sheets	Immunodeficient mice 8 weeks	Dentin-pulp like tissues and cemtum - periodontal complexes (cementum, PDL fibers and blood vessels) for both TDM and scaffolds with DFSC sheets
Lee 2014 et al.,[30]	PCL/HA 3D printed scaffolds with 100-300mm transverse microchannels, loaded with PLGA microspheres with rh-AM, CTGF and BMP2	Cell seeding with DPSCs	Harlan mice 4 weeks	DPSC-seeded multiphase scaffolds resulted in collagen fibers with PDL-like morphology that inserted aligned into new bone tissue and dentin/cementum-like constructs
Chen 2016 et al.,[31]	GelMA/nHA microgel arrays through photocrosslinking	No cells	BALB/c-nude mice 8 weeks	PDL-like tissue and blood vessels formed in the control, 1%, and 2% nHA groups
Varoni 2018 et al.,[32]	Chitosan- GEN- trilayer scaffold based on different molecular weight chitosan coated with fibrin gel	hPDL for the PDL interface, hGF cells for the gingival, and hOB cells for the bone	Nude immunedeficient mice (Athymic Nude-Foxn1nu) 6 weeks	Similar findings for all scaffolds: formation of connective tissue with newly formed vessels and dense mineralized matrix. Test scaffolds were richer in cells
Liao 2020 et al.,[33]	Mesoporous HA/chitosan composite scaffolds (mHA/CS)	hPDLCs seeded root slices /scaffolds	Nude mice (BALB/c)	Thin cementum-like tissue attached to the root surfaces was formed only in the mHA/CS-rhAM scaffolds and no

	loaded with rhAM	constructs wrapped with ePTFE	8 weeks	bone formation at any group. Fibrous tissue in parallel with the root slices, not attached in the unloaded scaffolds.
Zhao 2020 et al.,[24]	Simvastatin (SIM) -loaded PCL membrane scaffolds placed inbetween root dentin and ceramic bovine bone (CBB)	Periodontal ligament stem cells (PDLSCs)	Athymic nude rat 8 weeks	Ectopic cementum-like tissue formation on root dentin surface with organized collagenous tissue in close proximity to the cementum-like tissue
Yu 2022 et al.,[34]	Bilayer construct: IMC scaffold with CGF	N/A	Immunodeficient mice 8 weeks	Only the biomimetically constructed CGF/IMC scaffold showed new hard and soft fiber-like tissue formation

Supplementary Table S3. In vivo studies evaluating scaffolds for PDL regeneration employing other models than periodontal defect and subcutaneous placement model.

Study	Scaffold type	Cells	In vivo animal model	Evaluation time	Major findings
Wei 2013 et al.,[8]	HA/TCP scaffolds	PDLSCs and DPSCs	Bio-root implantation, root-shaped jawbone implant socket	Miniature pigs 6 months after implantation and 6 months after crown placement	Bone formation for all groups and PDL-like tissue along a dentin-like matrix structure for the cell-sheet wrapped scaffolds. Significant improvement of bone volume in the cell-sheet groups after crown placement and growth of the PDL tissue
Chen 2015 et al.,[35]	PLGA/Gelatin electrospun scaffolds (APES) with DPEM and TDM	DFSCs	Root-shaped jawbone implant sockets created on the location of 2nd premolars extracted sockets	Miniature swine pigs 12 weeks	DFSCs loaded composite (APES / TDM / DPEM) constructs yielded the formation of tooth root-like structures (cellular cementum and PDL-like tissues)
Chen 2016 et al.,[31]	Electrospun multiphasic scaffold of PCL, type I COL, and PEG-stabilized ACP nanoparticles loaded with rhCEMP	No cells	Calvaria defects	Wistar rats 4 and 8 weeks	Protein-releasing acellular biomaterial scaffold resulted in cementum-like tissue formation rich in cement like cells, but less bone formation. Island-like new bone was distributed discretely in rhCEMP1 loaded scaffolds (3)
Kim 2016 et al.,[36]	Electrospun PCL/gelatin directionally oriented nanofiber membrane	PDLCs under static and dynamic loading conditions	Intrabony defects in maxilla created after the extraction of incisors and subsequent replantation along with the membranes	Sprague-Dawley rats 4 weeks	Bone formation and PDL regeneration when implantation took place in remained PDL in the extraction socket. When PDL was removed, PDL regenerated without functional arrangement

Jiang 2020 et al.,[37]	PLGA scaffold loaded with pFGF-2 through electrospinning	No cells	Replantation of extracted anterior teeth	Beagle dogs 4 weeks after replantation	Immediate replantation group: well-organized PDL without root surface resorption. Delayed replantation PLGA scaffold group: root surface resorption. PLGA/ pFGF-2 scaffold group: PDL-like tissues with limited root resorption
Liu 2021 et al.,[38] gingipain…)	Hydrogel based on PEG-DA, DTT, and a novel functional peptide module (PEGPD scaffold)	Scaffolds loaded with SDF-1	Experimental periodontitis model: hydrogel injected buccally and palatally of maxillary 2nd molars	Wistar rats 4 weeks	In situ periodontal bone regeneration. Similar results for the PEGPD@SDF-1 and control group and similar periodontal ligament (PDL) arrangement

References

1. Yang, X.; Ma, Y.; Guo, W.; Yang, B.; Tian, W. Stem cells from human exfoliated deciduous teeth as an alternative cell source in bio-root regeneration. *Theranostics* **2019**, *9*, 2694–2711, doi:10.7150/thno.31801.
2. Washio, K.; Iwata, T.; Mizutani, M.; Ando, T.; Yamato, M.; Okano, T.; Ishikawa, I. Assessment of cell sheets derived from human periodontal ligament cells: A pre-clinical study. *Cell Tissue Res.* **2010**, *341*, 397–404, doi:10.1007/s00441-010-1009-1.
3. Xie, H.; Liu, H. A Novel Mixed-Type Stem Cell Pellet for Cementum/Periodontal Ligament–Like Complex. *J. Periodontol.* **2012**, *83*, 805–815, doi:10.1902/jop.2011.110267.
4. Ji, K.; Liu, Y.; Lu, W.; Yang, F.; Yu, J.; Wang, X.; Ma, Q.; Yang, Z.; Wen, L.; Xuan, K. Periodontal tissue engineering with stem cells from the periodontal ligament of human retained deciduous teeth. *J. Periodontal Res.* **2013**, *48*, 105–116, doi:10.1111/j.1600-0765.2012.01509.x.
5. Wei, F.; Qu, C.; Song, T.; Ding, G.; Fan, Z.; Liu, D.; Liu, Y.; Zhang, C.; Shi, S.; Wang, S. Vitamin C treatment promotes mesenchymal stem cell sheet formation and tissue regeneration by elevating telomerase activity. *J. Cell. Physiol.* **2012**, *227*, 3216–3224, doi:10.1002/jcp.24012.
6. Yang, B.; Chen, G.; Li, J.; Zou, Q.; Xie, D.; Chen, Y.; Wang, H.; Zheng, X.; Long, J.; Tang, W.; et al. Tooth root regeneration using dental follicle cell sheets in combination with a dentin matrix - based scaffold. *Biomaterials* **2012**, *33*, 2449–2461, doi:10.1016/j.biomaterials.2011.11.074.
7. Guo, S.; Guo, W.; Yi, D.; Gong, J.; Zou, Q.; Xie, D.; Chen, Y.; Wu, Y.; Tian, W. Comparative study of human dental follicle cell sheets and periodontal ligament cell sheets for periodontal tissue regeneration. *Cell Transplant.* **2013**, *22*, 1061–1073, doi:10.3727/096368912X656036.
8. Wei, F.; Song, T.; Ding, G.; Xu, J.; Liu, Y.; Liu, D.; Fan, Z.; Zhang, C.; Shi, S.; Wang, S. Functional tooth restoration by allogeneic mesenchymal stem cell-based bio-root regeneration in swine. *Stem Cells Dev.* **2013**, *22*, 1752–1762, doi:10.1089/scd.2012.0688.
9. Liu, J.; Wang, L.; Liu, W.; Li, Q.; Jin, Z.; Jin, Y. Dental follicle cells rescue the regenerative capacity of periodontal ligament stem cells in an inflammatory microenvironment. *PLoS One* **2014**, *9*, doi:10.1371/journal.pone.0108752.

10. Q., X.; B., L.; L., Y.; Z., D.; H., Z.; H., W.; J., S.; S., G.; Y., J. Combination of platelet-rich plasma within periodontal ligament stem cell sheets enhances cell differentiation and matrix production. *J. Tissue Eng. Regen. Med.* **2017**, *11*, 627–636.
11. Guo, W.; He, Y.; Tang, X.; Chen, G.; Shi, H.; Gong, K.; Zhou, J.; Wen, L.; Jin, Y. Scaffold-free cell pellet transplantations can be applied to periodontal regeneration. *Cell Transplant.* **2014**, *23*, 181–194, doi:10.3727/096368912X662426.
12. Gao, H.; Li, B.; Zhao, L.; Jin, Y. Influence of nanotopography on periodontal ligament stem cell functions and cell sheet based periodontal regeneration. *Int. J. Nanomedicine* **2015**, *10*, 4009–4027, doi:10.2147/IJN.S83357.
13. Zhang, H.; Liu, S.; Zhu, B.; Xu, Q.; Ding, Y.; Jin, Y. Composite cell sheet for periodontal regeneration: crosstalk between different types of MSCs in cell sheet facilitates complex periodontal-like tissue regeneration. *Stem Cell Res. Ther.* **2016**, *7*, 1–15, doi:10.1186/s13287-016-0417-x.
14. Panduwawala, C.P.; Zhan, X.; Dissanayaka, W.L.; Samaranayake, L.P.; Jin, L.; Zhang, C. In vivo periodontal tissue regeneration by periodontal ligament stem cells and endothelial cells in three-dimensional cell sheet constructs. *J. Periodontal Res.* **2017**, *52*, 408–418, doi:10.1111/jre.12405.
15. Yu, Y.; Bi, C.S.; Wu, R.X.; Yin, Y.; Zhang, X.Y.; Lan, P.H.; Chen, F.M. Effects of short-term inflammatory and/or hypoxic pretreatments on periodontal ligament stem cells: in vitro and in vivo studies. *Cell Tissue Res.* **2016**, *366*, 311–328, doi:10.1007/s00441-016-2437-3.
16. Wang, Z.S.; Feng, Z.H.; Wu, G.F.; Bai, S.Z.; Dong, Y.; Chen, F.M.; Zhao, Y.M. The use of platelet-rich fibrin combined with periodontal ligament and jaw bone mesenchymal stem cell sheets for periodontal tissue engineering. *Sci. Rep.* **2016**, *6*, 1–15, doi:10.1038/srep28126.
17. Gao, Z.H.; Hu, L.; Liu, G.L.; Wei, F.L.; Liu, Y.; Liu, Z.H.; Fan, Z.P.; Zhang, C.M.; Wang, J.S.; Wang, S.L. Bio-root and implant-based restoration as a tooth replacement alternative. *J. Dent. Res.* **2016**, *95*, 642–649, doi:10.1177/0022034516639260.
18. Feng, G.; Wu, Y.; Yu, Y.; Huang, L.; An, S.; Hu, B.; Luo, J.; Song, J. Periodontal ligament-like tissue regeneration with drilled porous decalcified dentin matrix sheet composite. *Oral Dis.* **2018**, *24*, 429–441, doi:10.1111/odi.12734.
19. Zhang, J.C.; Song, Z.C.; Xia, Y.R.; Shu, R. Extracellular matrix derived from periodontal ligament cells maintains their stemness and enhances redifferentiation via the wnt pathway. *J. Biomed. Mater. Res. - Part A* **2018**, *106*, 272–284, doi:10.1002/jbm.a.36227.
20. Washio, K.; Tsutsumi, Y.; Tsumanuma, Y.; Yano, K.; Srithanyarat, S.S.; Takagi, R.; Ichinose, S.; Meinzer, W.; Yamato, M.; Okano, T.; et al. In Vivo Periodontium Formation Around Titanium Implants Using Periodontal Ligament Cell Sheet. *Tissue Eng. - Part A* **2018**, *24*, 1273–1282, doi:10.1089/ten.tea.2017.0405.
21. Hu, L.; Zhao, B.; Gao, Z.; Xu, J.; Fan, Z.; Zhang, C.; Wang, J.; Wang, S. Regeneration characteristics of different dental derived stem cell sheets. *J. Oral Rehabil.* **2020**, *47*, 66–72, doi:10.1111/joor.12839.
22. Yang, X.; Xiong, X.; Zhou, W.; Feng, G.; Zhang, Y.; Dai, H.; Zhou, J. Effects of human urine-derived stem cells on the cementogenic differentiation of indirectly-cocultured periodontal ligament stem cells. *Am. J. Transl. Res.* **2020**, *12*, 361–378.
23. Li, H.; Zhou, J.; Zhu, M.; Ying, S.; Li, L.; Chen, D.; Li, J.; Song, J. Low-intensity pulsed ultrasound promotes the formation of periodontal ligament stem cell sheets and ectopic periodontal tissue regeneration. *J. Biomed. Mater. Res. - Part A* **2021**, *109*, 1101–1112, doi:10.1002/jbm.a.37102.
24. Zhao, B.; Chen, J.; Zhao, L.; Deng, J.; Li, Q. A simvastatin-releasing scaffold with periodontal ligament stem cell sheets for periodontal regeneration. *J. Appl. Biomater. Funct. Mater.* **2020**, *18*, doi:10.1177/2280800019900094.
25. Park, J.Y.; Park, C.H.; Yi, T.; Kim, S.N.; Iwata, T.; Yun, J.H. RhBMP-2 pre-treated human periodontal ligament stem cell sheets regenerate a mineralized layer mimicking dental cementum. *Int. J. Mol. Sci.* **2020**, *21*, 1–16, doi:10.3390/ijms21113767.
26. Meng, H.; Hu, L.; Zhou, Y.; Ge, Z.; Wang, H.; Wu, C.T.; Jin, J. *A Sandwich Structure of Human Dental Pulp Stem Cell Sheet, Treated Dentin Matrix, and Matrigel for Tooth Root Regeneration*; 2020; Vol. 29; ISBN 1391002636.
27. Raju, R.; Oshima, M.; Inoue, M.; Morita, T.; Huijiao, Y.; Waskitho, A.; Baba, O.; Inoue, M.; Matsuka, Y. Three-dimensional periodontal tissue regeneration using a bone-ligament complex cell sheet. *Sci. Rep.* **2020**, *10*, 1–16, doi:10.1038/s41598-020-58222-0.

28. Park, C.H.; Rios, H.F.; Jin, Q.; Bland, M.E.; Flanagan, C.L.; Hollister, S.J.; Giannobile, W. V. Biomimetic hybrid scaffolds for engineering human tooth-ligament interfaces. *Biomaterials* **2010**, *31*, 5945–5952, doi:10.1016/j.biomaterials.2010.04.027.
29. Vaquette, C.; Fan, W.; Xiao, Y.; Hamlet, S.; Hutmacher, D.W.; Ivanovski, S. A biphasic scaffold design combined with cell sheet technology for simultaneous regeneration of alveolar bone/periodontal ligament complex. *Biomaterials* **2012**, *33*, 5560–5573, doi:10.1016/j.biomaterials.2012.04.038.
30. Lee, C.H.; Hajibandeh, J.; Suzuki, T.; Fan, A.; Shang, P.; Mao, J.J. Three-dimensional printed multiphase scaffolds for regeneration of periodontium complex. *Tissue Eng. - Part A* **2014**, *20*, 1342–1351, doi:10.1089/ten.tea.2013.0386.
31. Chen, X.; Liu, Y.; Miao, L.; Wang, Y.; Ren, S.; Yang, X.; Hu, Y.; Sun, W. Controlled release of recombinant human cementum protein 1 from electrospun multiphasic scaffold for cementum regeneration. *Int. J. Nanomedicine* **2016**, *11*, 3145–3158, doi:10.2147/IJN.S104324.
32. Varoni, E.M.; Vijayakumar, S.; Canciani, E.; Cochis, A.; De Nardo, L.; Lodi, G.; Rimondini, L.; Cerruti, M. Chitosan-Based Trilayer Scaffold for Multitissue Periodontal Regeneration. *J. Dent. Res.* **2018**, *97*, 303–311, doi:10.1177/0022034517736255.
33. Liao, Y.; Li, H.; Shu, R.; Chen, H.; Zhao, L.; Song, Z.; Zhou, W. Mesoporous Hydroxyapatite/Chitosan Loaded With Recombinant-Human Amelogenin Could Enhance Antibacterial Effect and Promote Periodontal Regeneration. *Front. Cell. Infect. Microbiol.* **2020**, *10*, doi:10.3389/fcimb.2020.00180.
34. Yu, M.; Luo, D.; Qiao, J.; Guo, J.; He, D.; Jin, S.; Tang, L.; Wang, Y.; Shi, X.; Mao, J.; et al. A hierarchical bilayer architecture for complex tissue regeneration. *Bioact. Mater.* **2022**, *10*, 93–106, doi:10.1016/j.bioactmat.2021.08.024.
35. Chen, G.; Chen, J.; Yang, B.; Li, L.; Luo, X.; Zhang, X.; Feng, L.; Jiang, Z.; Yu, M.; Guo, W.; et al. Combination of aligned PLGA/Gelatin electrospun sheets, native dental pulp extracellular matrix and treated dentin matrix as substrates for tooth root regeneration. *Biomaterials* **2015**, *52*, 56–70, doi:10.1016/j.biomaterials.2015.02.011.
36. Kim, J.H.; Kang, M.S.; Eltohamy, M.; Kim, T.H.; Kim, H.W. Dynamic mechanical and nanofibrous topological combinatory cues designed for periodontal ligament engineering. *PLoS One* **2016**, *11*, 1–22, doi:10.1371/journal.pone.0149967.
37. Jiang, L.; Ding, Z.; Xia, S.; Liu, Y.; Lei, S.; Zhong, M.; Chen, X. Poly lactic-co-glycolic acid scaffold loaded with plasmid DNA encoding fibroblast growth factor-2 promotes periodontal ligament regeneration of replanted teeth. *J. Periodontal Res.* **2020**, *55*, 488–495, doi:10.1111/jre.12734.
38. Liu, S.; Wang, Y.N.; Ma, B.; Shao, J.; Liu, H.; Ge, S. Gingipain-Responsive Thermosensitive Hydrogel Loaded with SDF-1 Facilitates in Situ Periodontal Tissue Regeneration. *ACS Appl. Mater. Interfaces* **2021**, *13*, 36880–36893, doi:10.1021/acsami.1c08855.
